# Supplementary material for: Oxolane Ammonium Salts (Muscarine-Like)—Synthesis and Microbiological Activity
Source: Int J Mol Sci. 2024 Feb 17;25(4):2368. doi: 10.3390/ijms25042368 (PMC10889796; doi:10.3390/ijms25042368)
Supplement: Supplementary file 1 [file ijms-25-02368-s001.zip › ijms-2855712-supplementary.pdf]

Supporting information

Oxolane ammonium salts (Muscarine-like) – Synthesis and Microbiological Activity

Patrycja Bogdanowicz[1], Janusz Madaj[1], Piotr Szweda[2], Artur Sikorski[1], Justyna Samaszko-Fiertek[1] and Barbara Dmochowska[1,*]
[1] Faculty of Chemistry, University of Gdansk, Wita Stwosza 63, 80-308 Gdansk, Poland
[2] Department of Pharmaceutical Technology and Biochemistry, Gdansk University of Technology, Gabriela Narutowicza Street 11/12, 80-233 Gdansk, Poland
***** Correspondence: basia.dmochowska@ug.edu.pl; Tel.: +48-58-523-50-69;

General procedures, analytical data and spectra of all new compounds

Contents

NMR spectra **S2-S41**

MS spectra **S42-S50**

IR spectra **S51-S60**

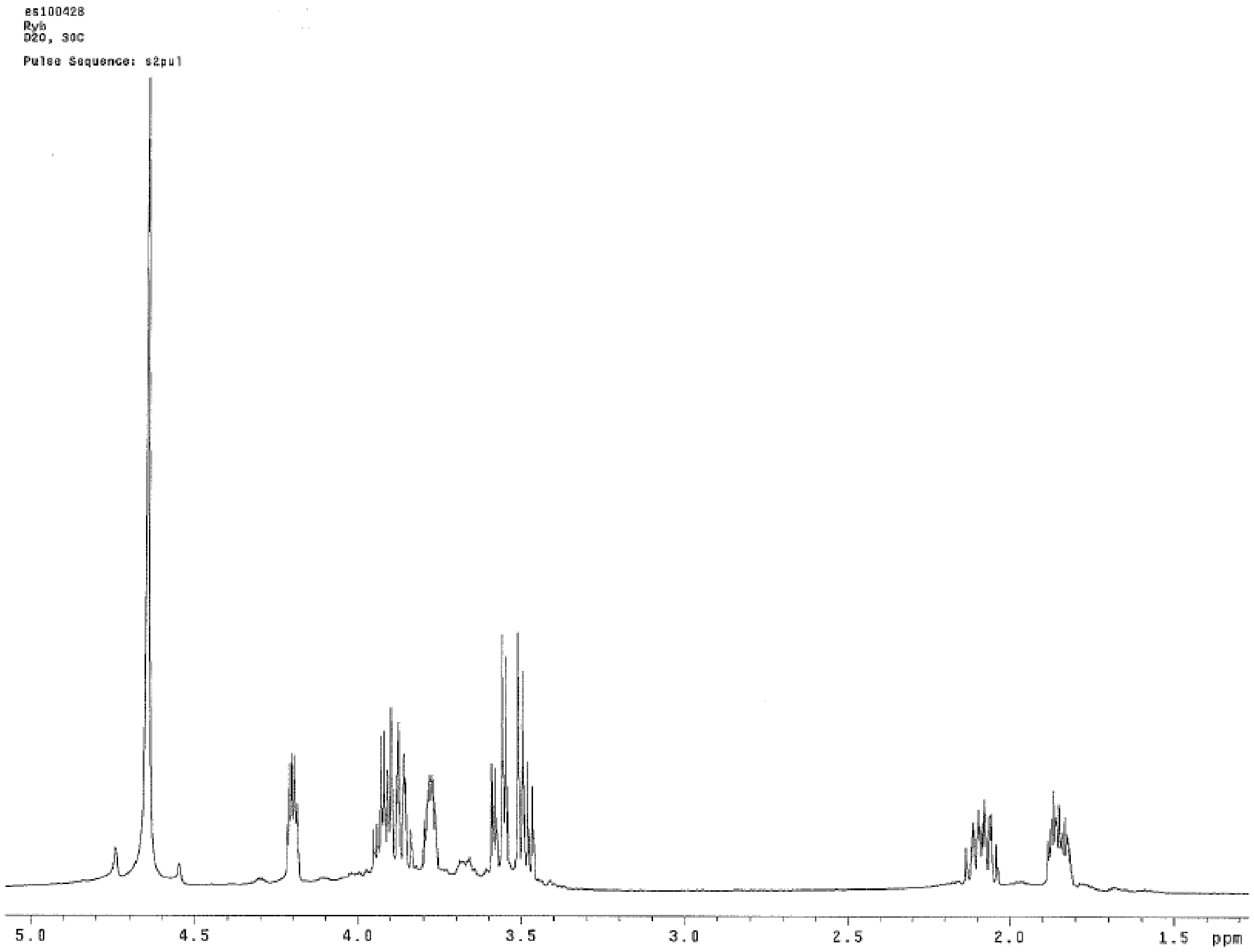

Figure S1. ^{1}H NMR spectrum (400 MHz, D_2O) of (2*R*,3*S*)-2-(hydroxymethyl)oxolan-3-ol (**3**).

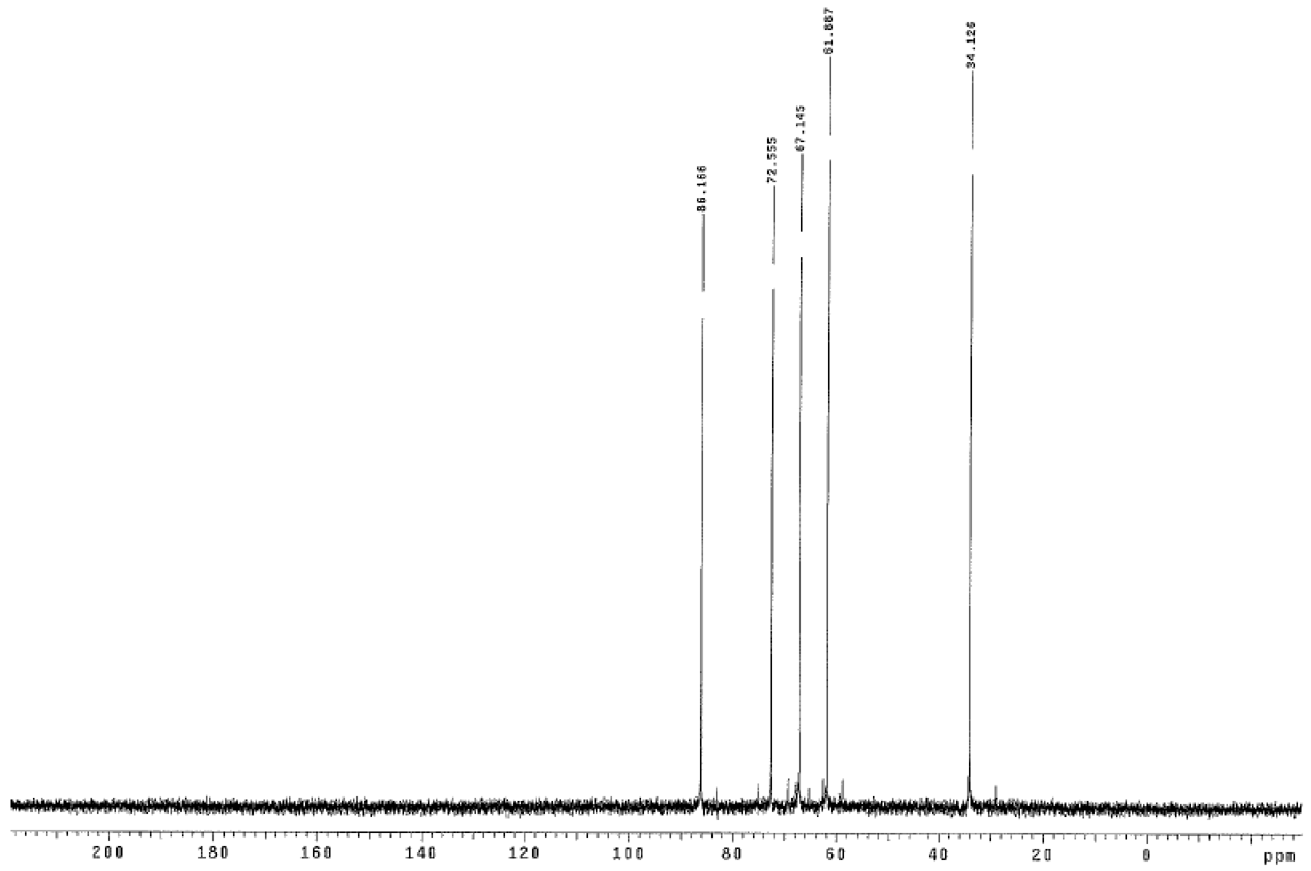

Figure S2. ^{13}C NMR spectrum (100 MHz, D_2O) of (2*R*,3*S*)-2-(hydroxymethyl)oxolan-3-ol (**3**).

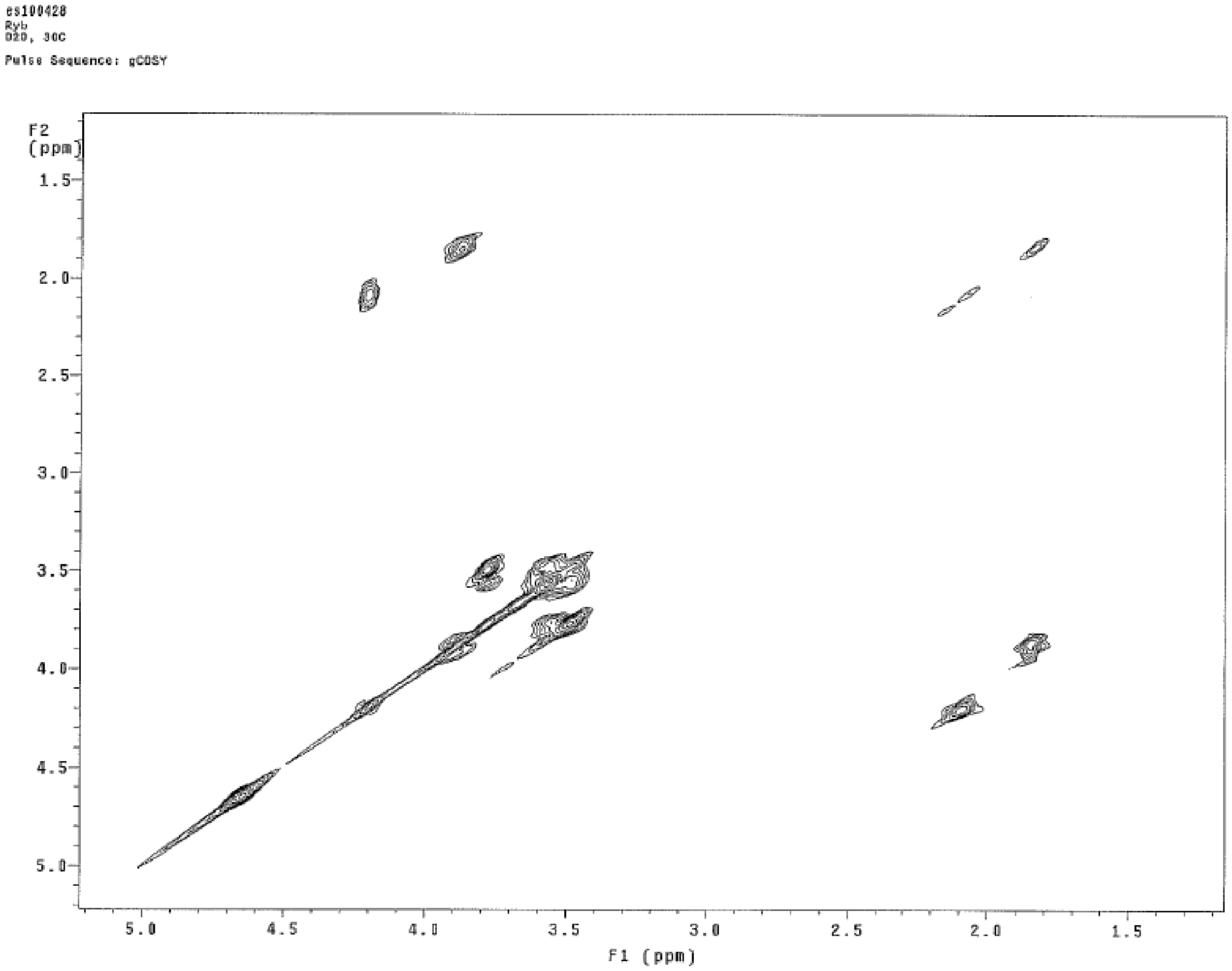

Figure S3. COSY spectrum of (2*R*,3*S*)-2-(hydroxymethyl)oxolan-3-ol (**3**).

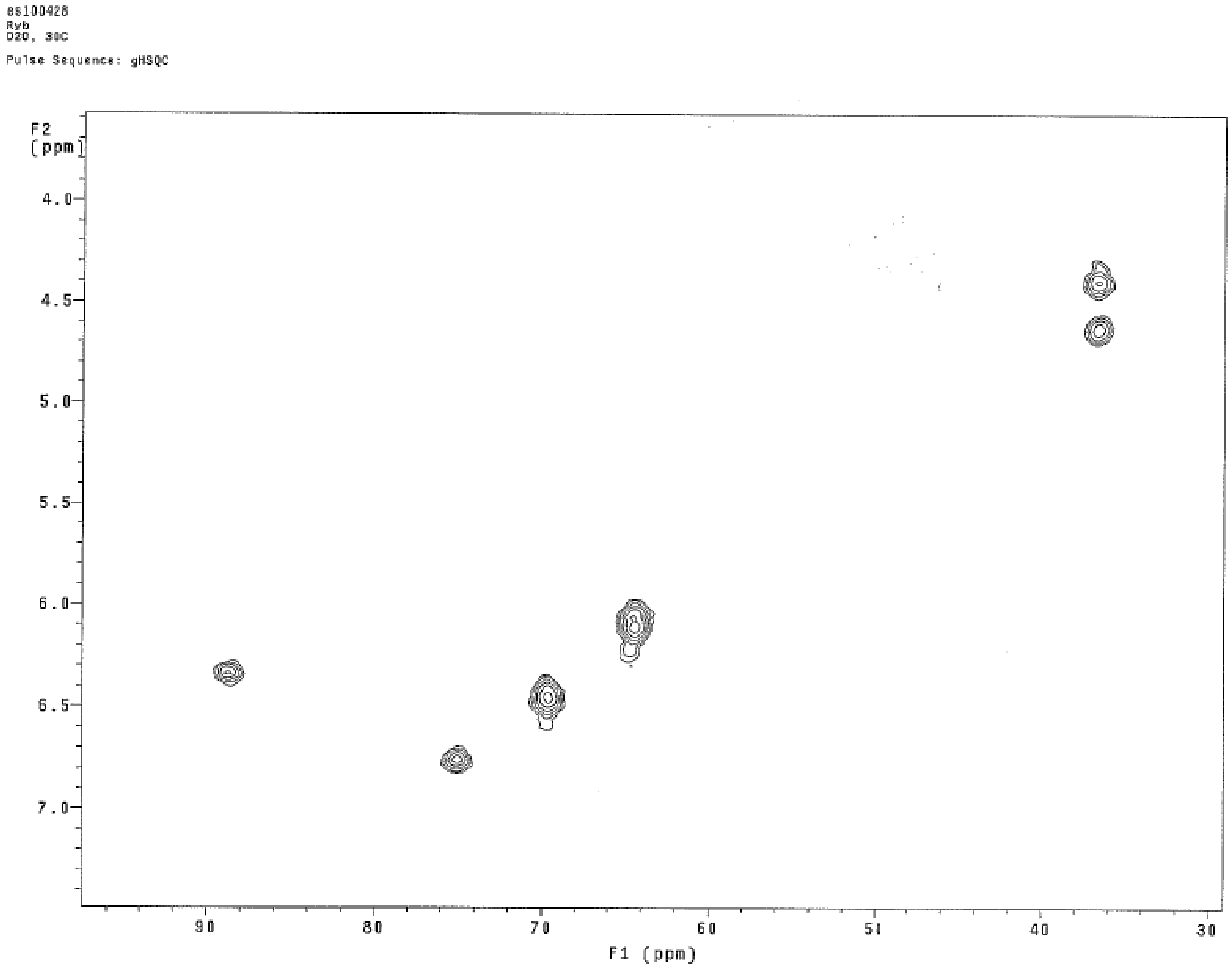

Figure S4. HSQC spectrum of (2*R*,3*S*)-2-(hydroxymethyl)oxolan-3-ol (**3**).

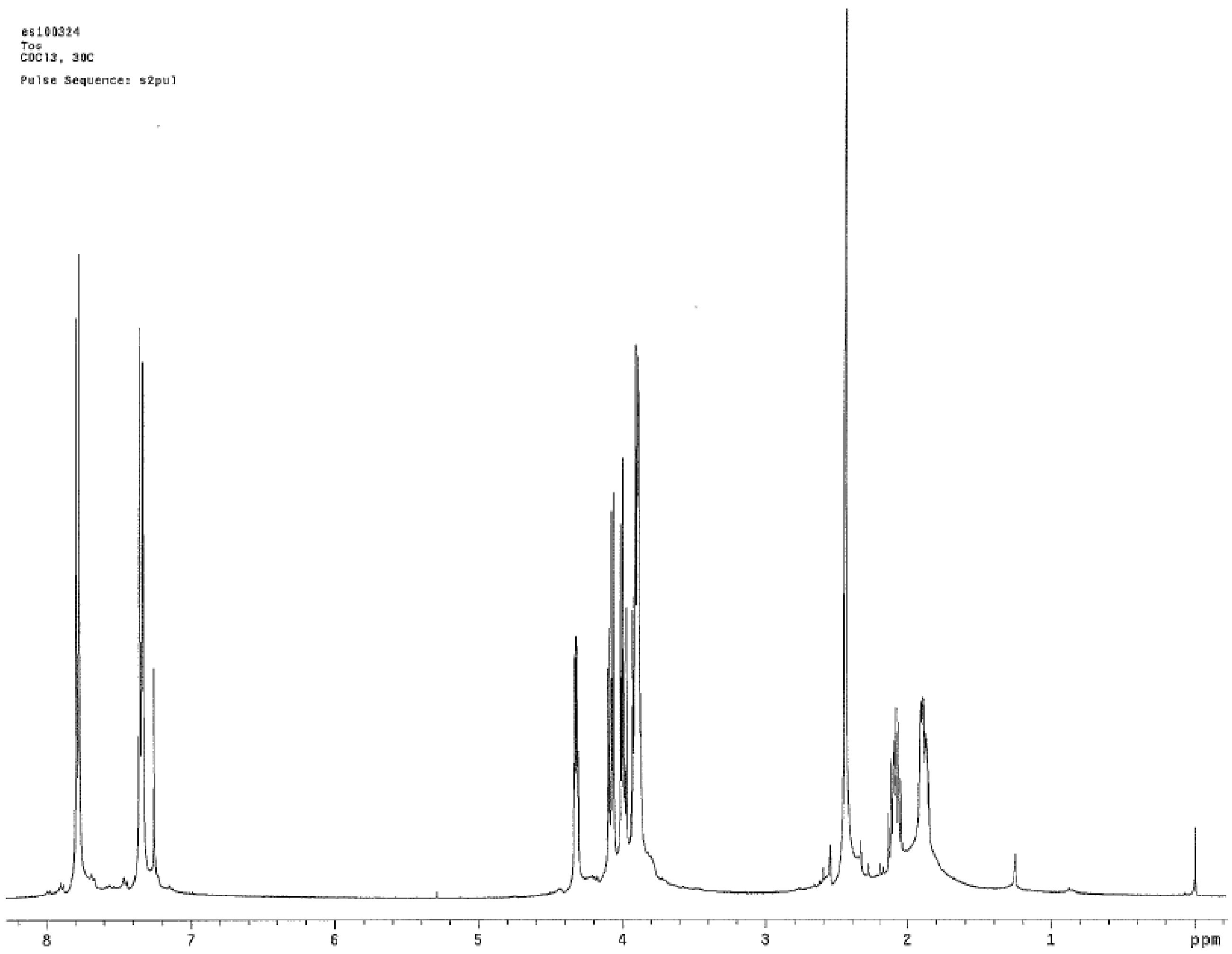

Figure S5. ^{1}H NMR spectrum (400 MHz, $CDCl_3$) of (2*R*,3*S*)-2-(*O*-tosylmethoxyl)oxolan-3-ol (**4**).

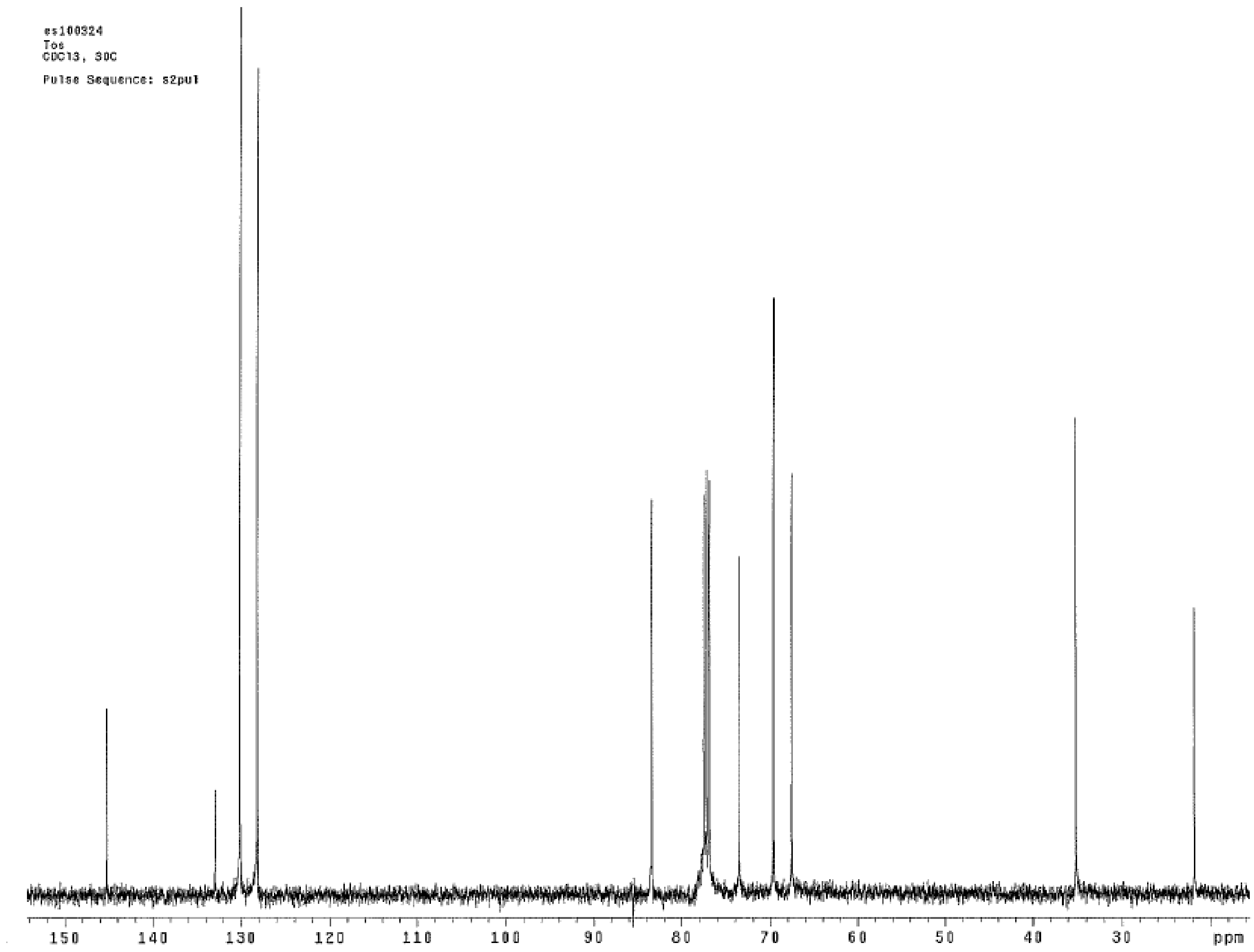

Figure S6. ^{13}C NMR spectrum (100 MHz, $CDCl_3$) of (2*R*,3*S*)-2-(*O*-tosylmethoxyl)oxolan-3-ol (**4**).

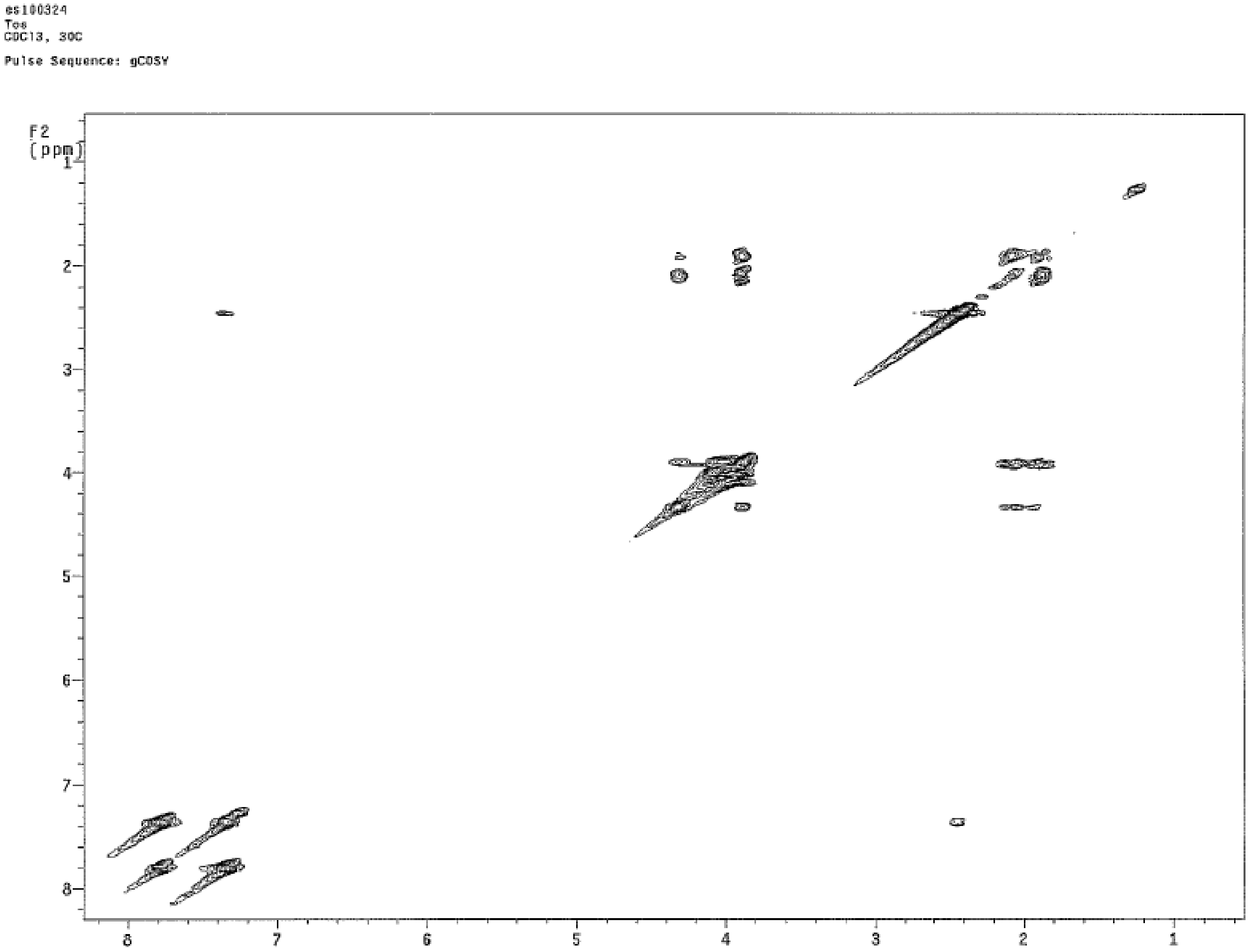

Figure S7. COSY spectrum of (2*R*,3*S*)-2-(*O*-tosylmethoxyl)oxolan-3-ol (**4**).

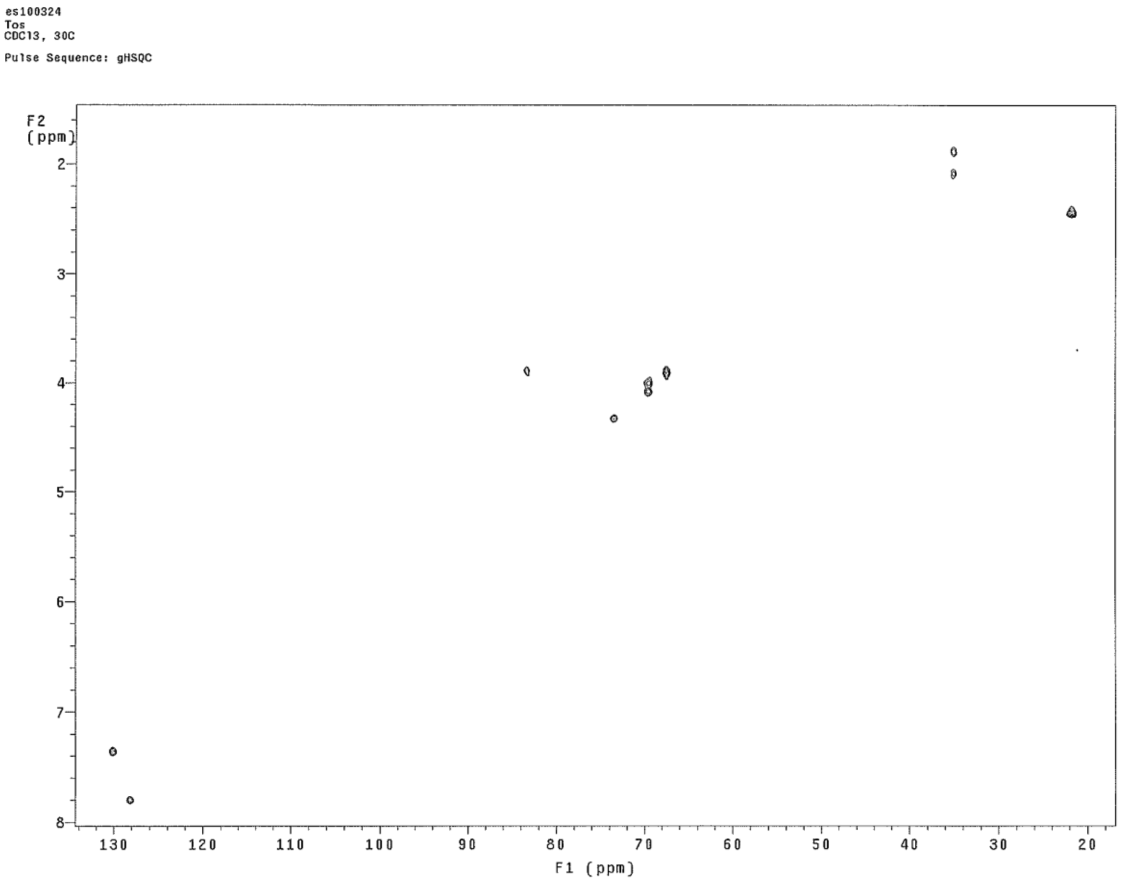

Figure S8. HSQC spectrum of (2*R*,3*S*)-2-(*O*-tosylmethoxyl)oxolan-3-ol (**4**).

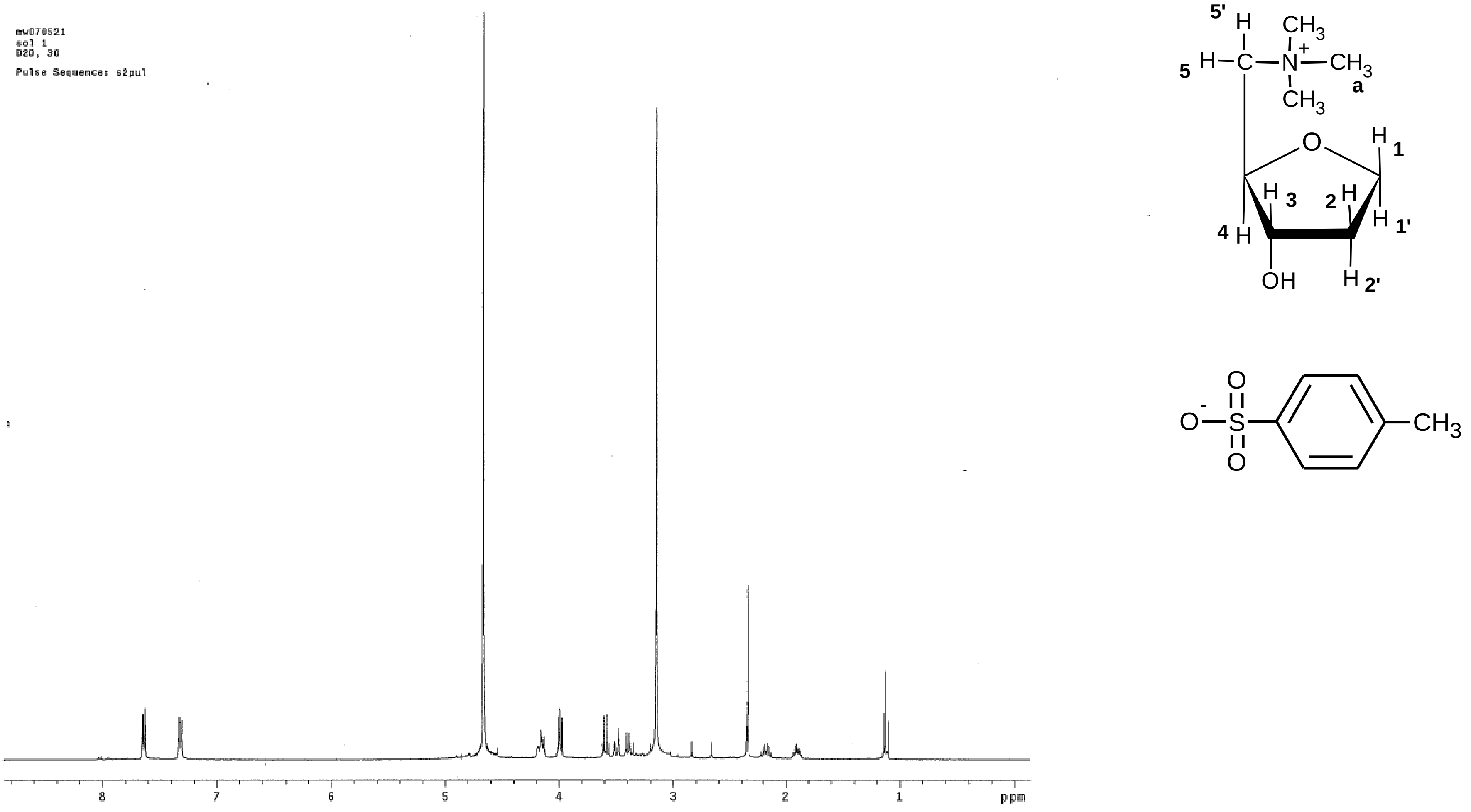

Figure S9. ^{1}H NMR spectrum (400 MHz, D_2O) of *N*-[(2*R*,3*S*)-(3-hydroxyoxolan-2-yl)methyl]-*N*,*N*,*N*-trimethylamonium tosylate (**5a**).

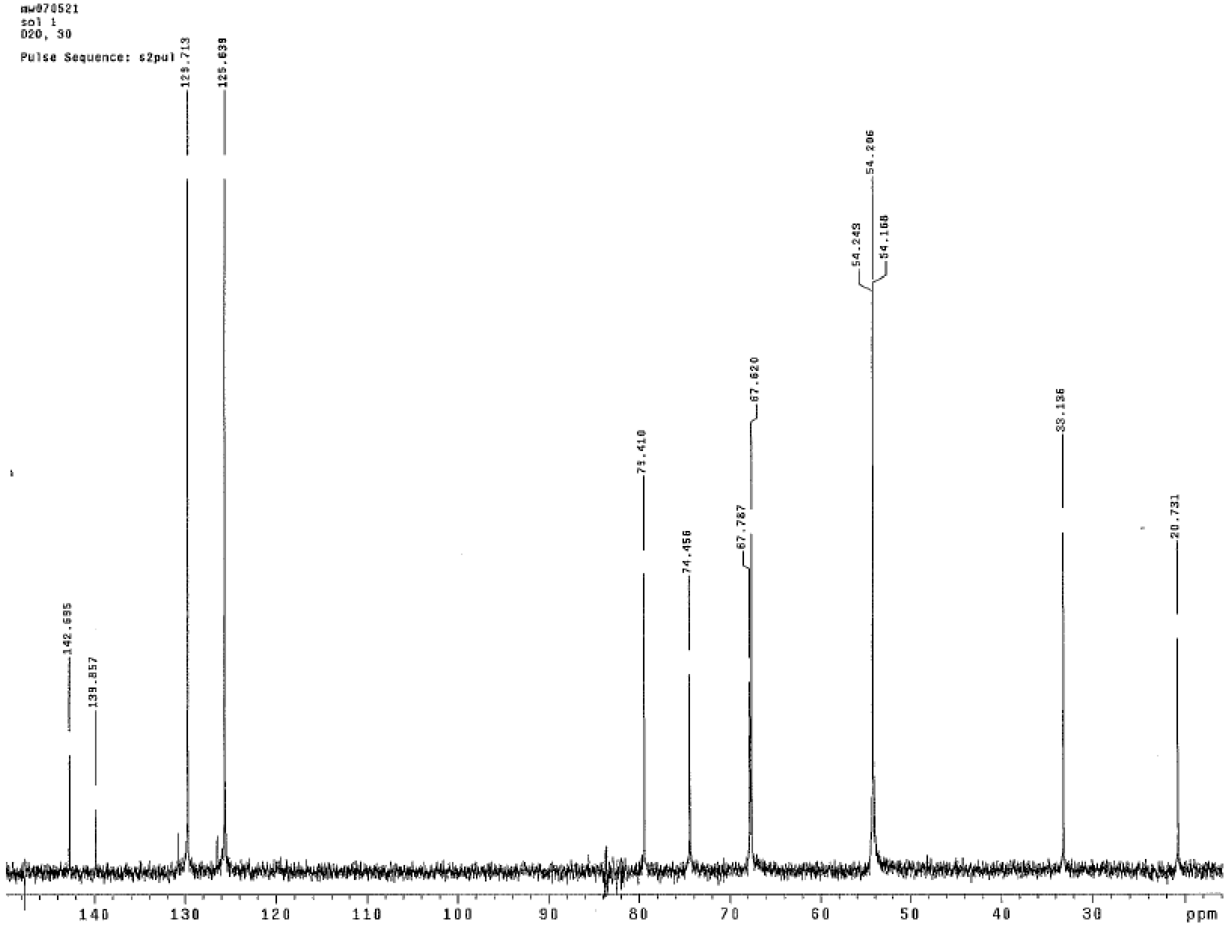

Figure S10. ^{13}C NMR spectrum (100 MHz, D_2O) of *N*-[(2*R*,3*S*)-(3-hydroxyoxolan-2-yl)methyl]-*N*,*N*,*N*-trimethylamonium tosylate (**5a**).

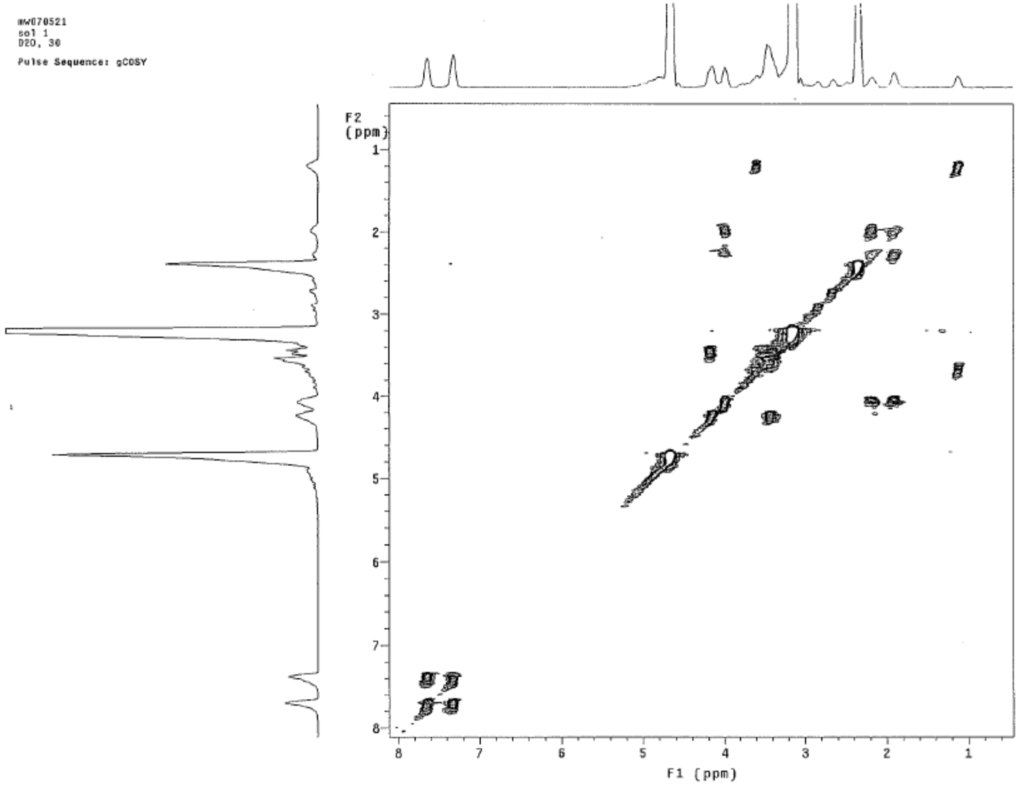

Figure S11. COSY spectrum of *N*-[(2*R*,3*S*)-(3-hydroxyoxolan-2-yl)methyl]-*N*,*N*,*N*-trimethylamonium tosylate (**5a**).

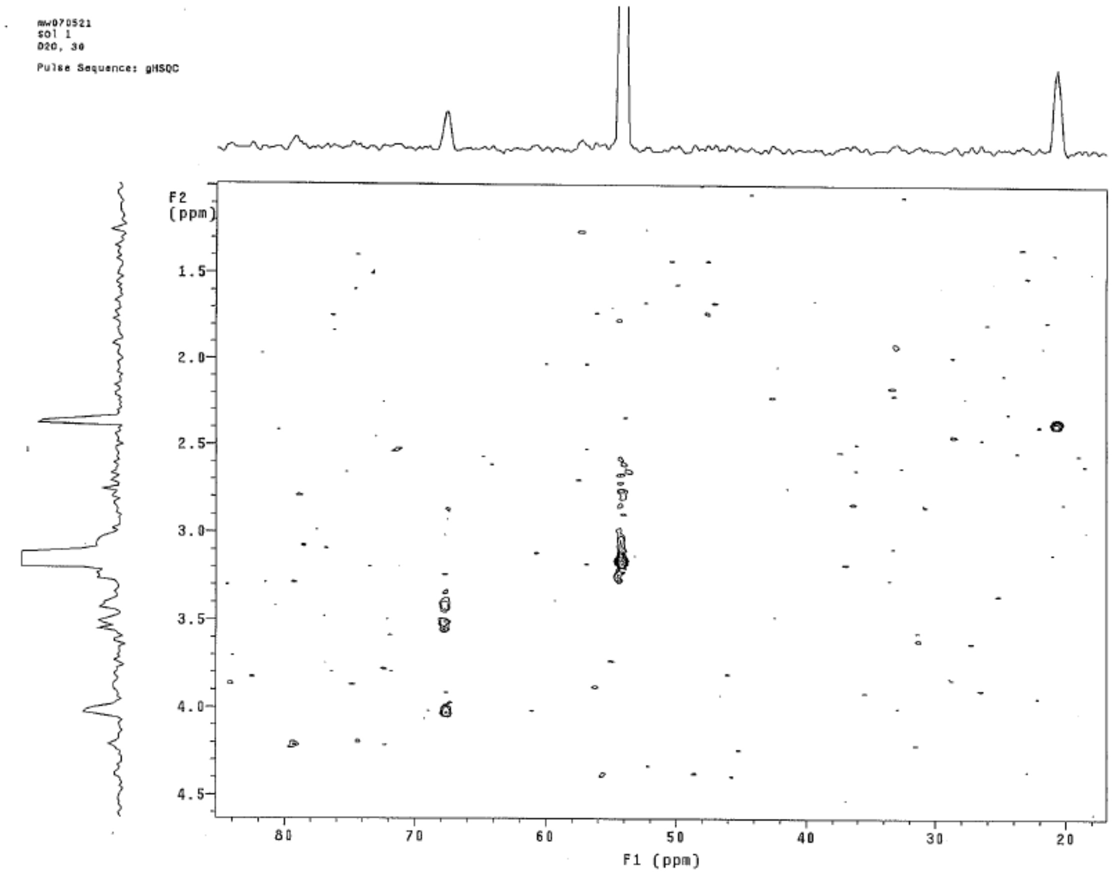

Figure S12. HSQC spectrum of *N*-[(2*R*,3*S*)-(3-hydroxyoxolan-2-yl)methyl]-*N*,*N*,*N*-trimethylamonium tosylate (**5a**).

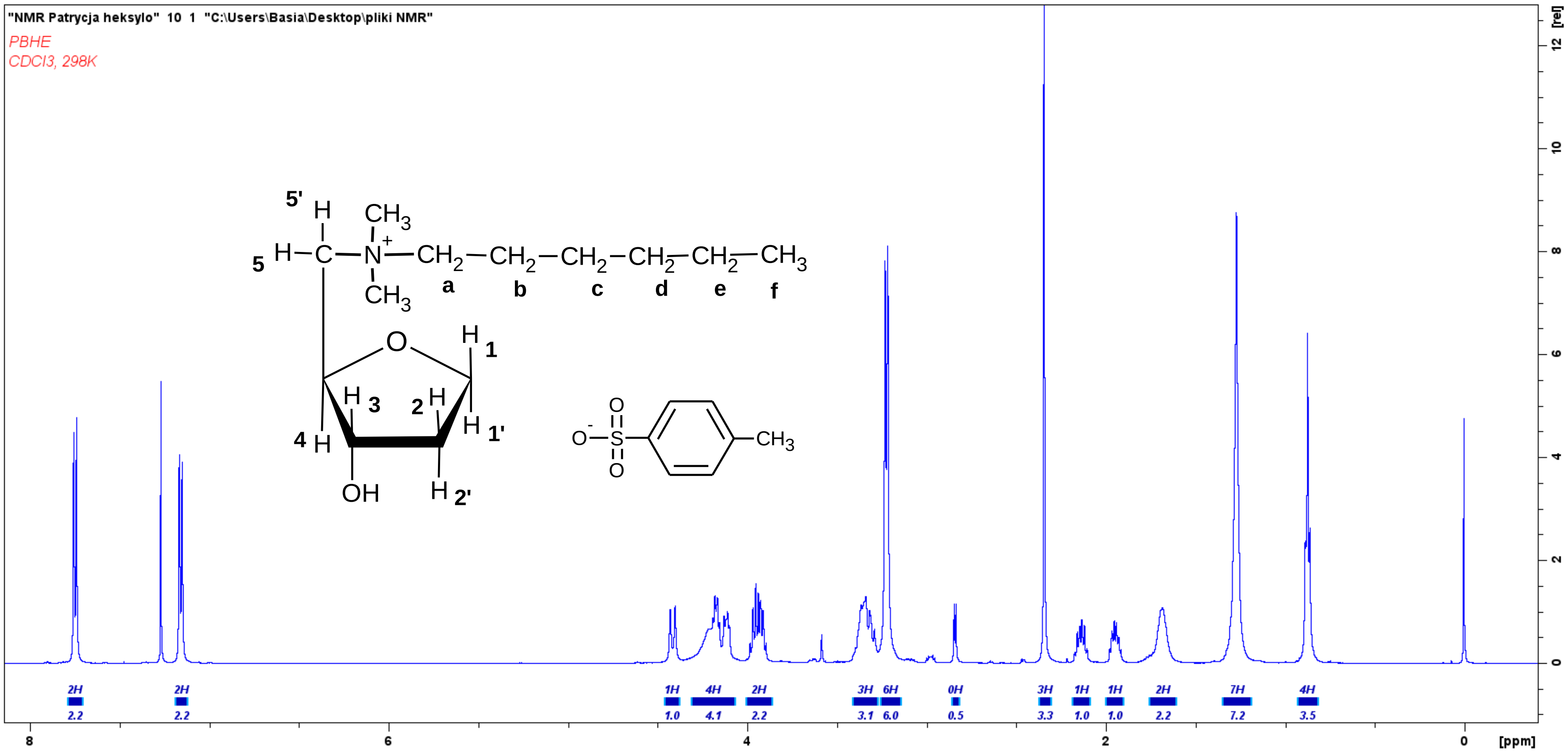

Figure S13. ^{1}H NMR spectrum (500 MHz, ~~D_2O~~ $CDCl_3$) of *N*-[(2*R*,3*S*)-(3-hydroxyoxolan-2-yl)methyl]-*N*-hexyl-*N*,*N*-dimethylammonium tosylate (**5b**).

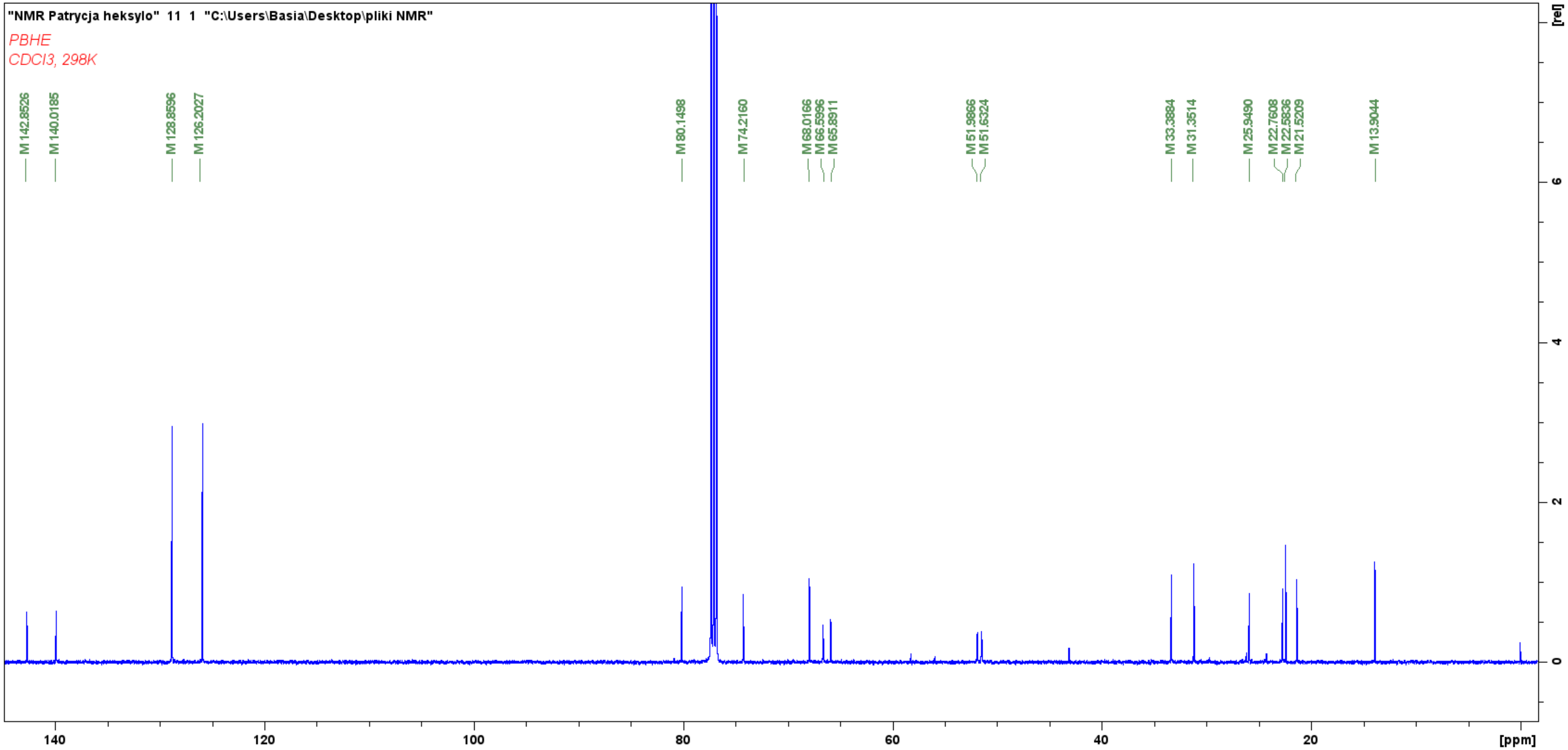

Figure S14. ^{13}C NMR spectrum (125 MHz, ~~D_2O~~ $CDCl_3$) of *N*-[(2*R*,3*S*)-(3-hydroxyoxolan-2-yl)methyl]-*N*-hexyl-*N*,*N*-dimethylammonium tosylate (**5b**).

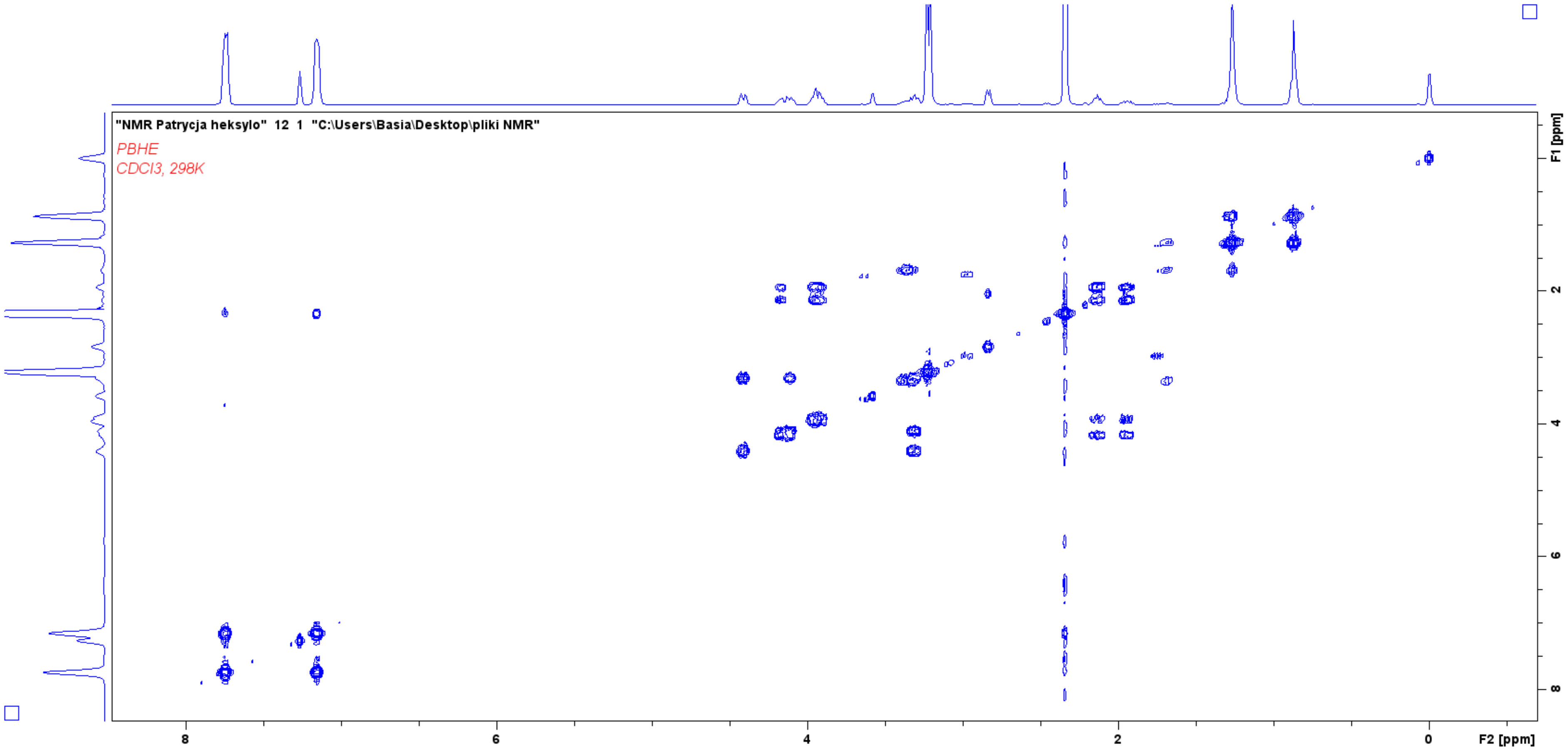

Figure S15. COSY spectrum of *N*-[(2*R*,3*S*)-(3-hydroxyoxolan-2-yl)methyl]-*N*-hexyl-*N*,*N*-dimethylammonium tosylate (**5b**).

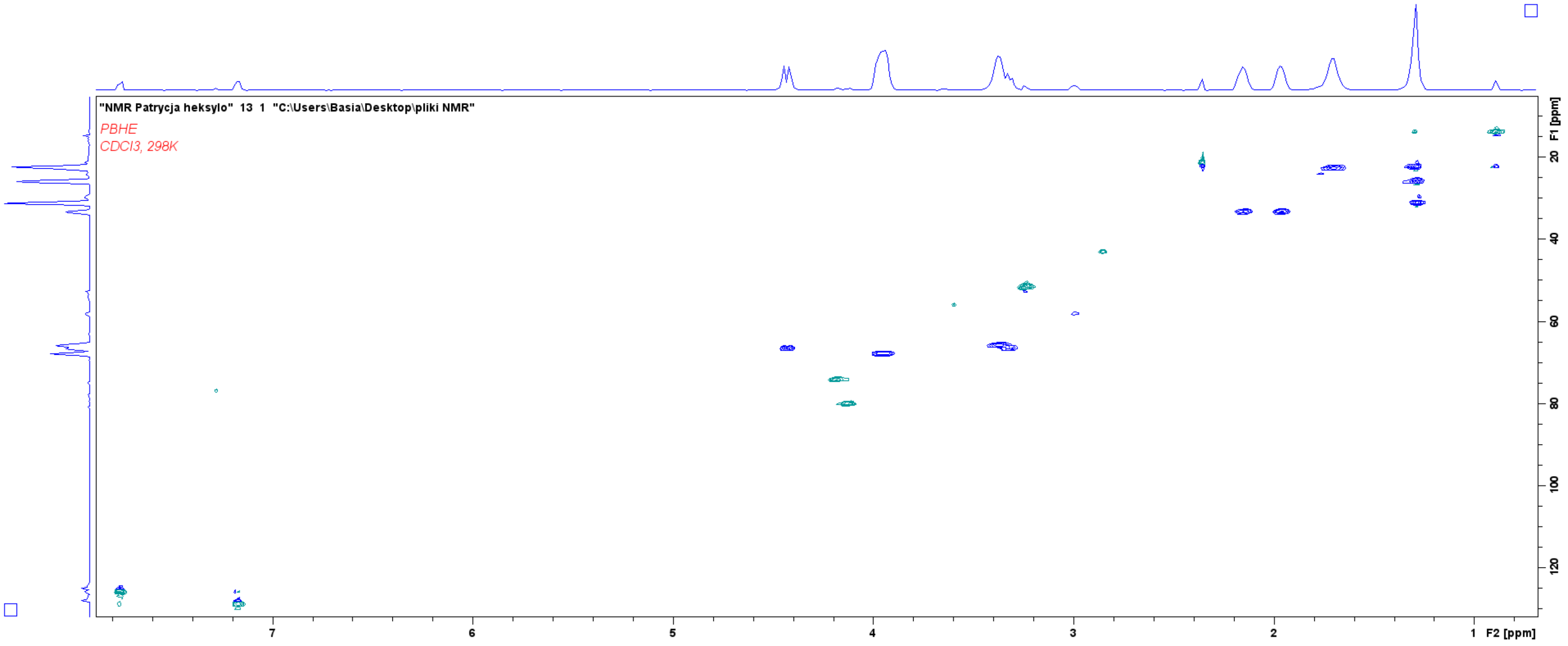

Figure S16. HSQC spectrum of *N*-[(2*R*,3*S*)-(3-hydroxyoxolan-2-yl)methyl]-*N*-hexyl-*N*,*N*-dimethylammonium tosylate (**5b**).

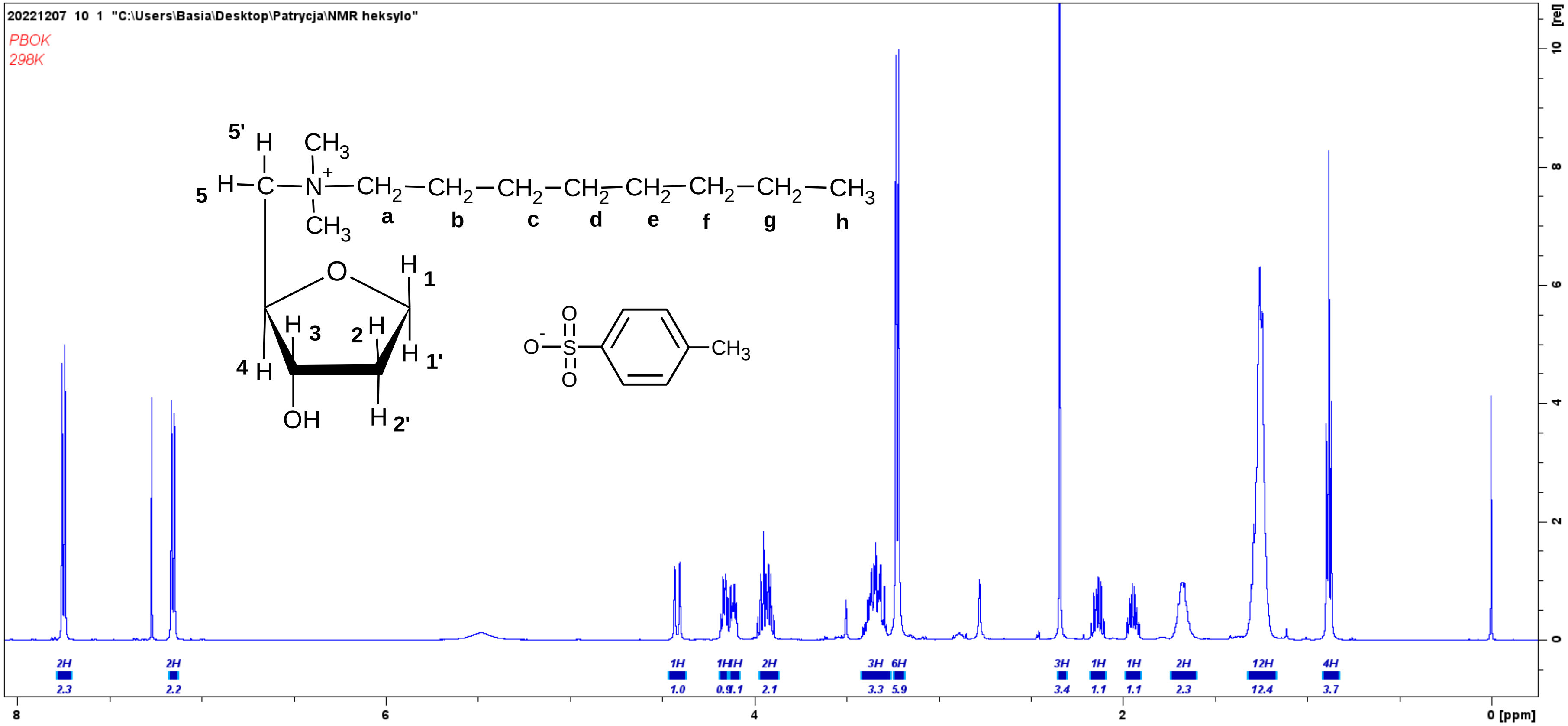

Figure S17. ^{1}H NMR spectrum (500 MHz, ~~D_2O~~ $CDCl_3$) of *N*-[(2*R*,3*S*)-(3-hydroxyoxolan-2-yl)methyl]-*N*,*N*-dimethyl-*N*-octylammonium tosylate (**5c**).

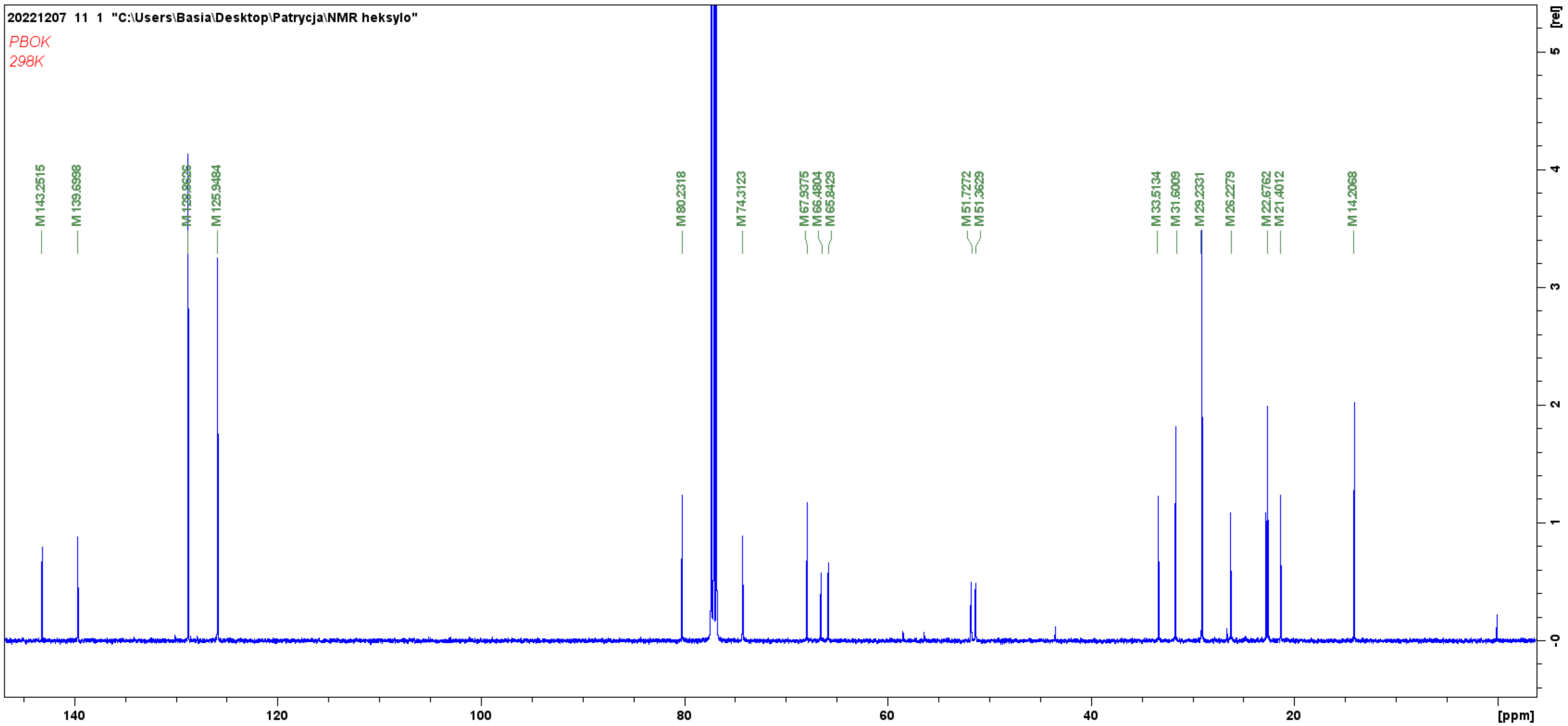

Figure S18. ^{13}C NMR spectrum (125 MHz, ~~D_2O~~ $CDCl_3$) of *N*-[(2*R*,3*S*)-(3-hydroxyoxolan-2-yl)methyl]-*N*,*N*-dimethyl-*N*-octylammonium tosylate (**5c**).

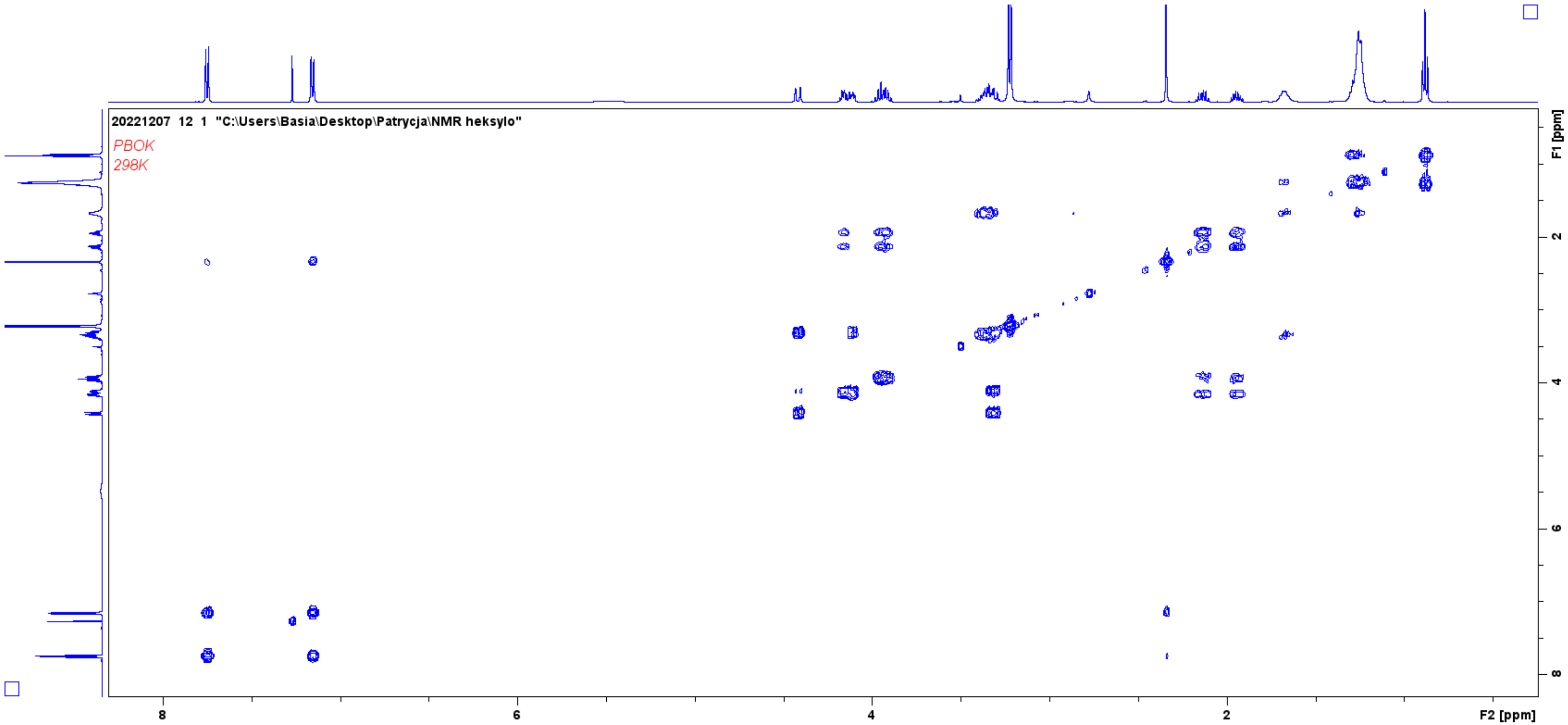

Figure S19. COSY spectrum of *N*-[(2*R*,3*S*)-(3-hydroxyoxolan-2-yl)methyl]-*N*,*N*-dimethyl-*N*-octylammonium tosylate (**5c**).

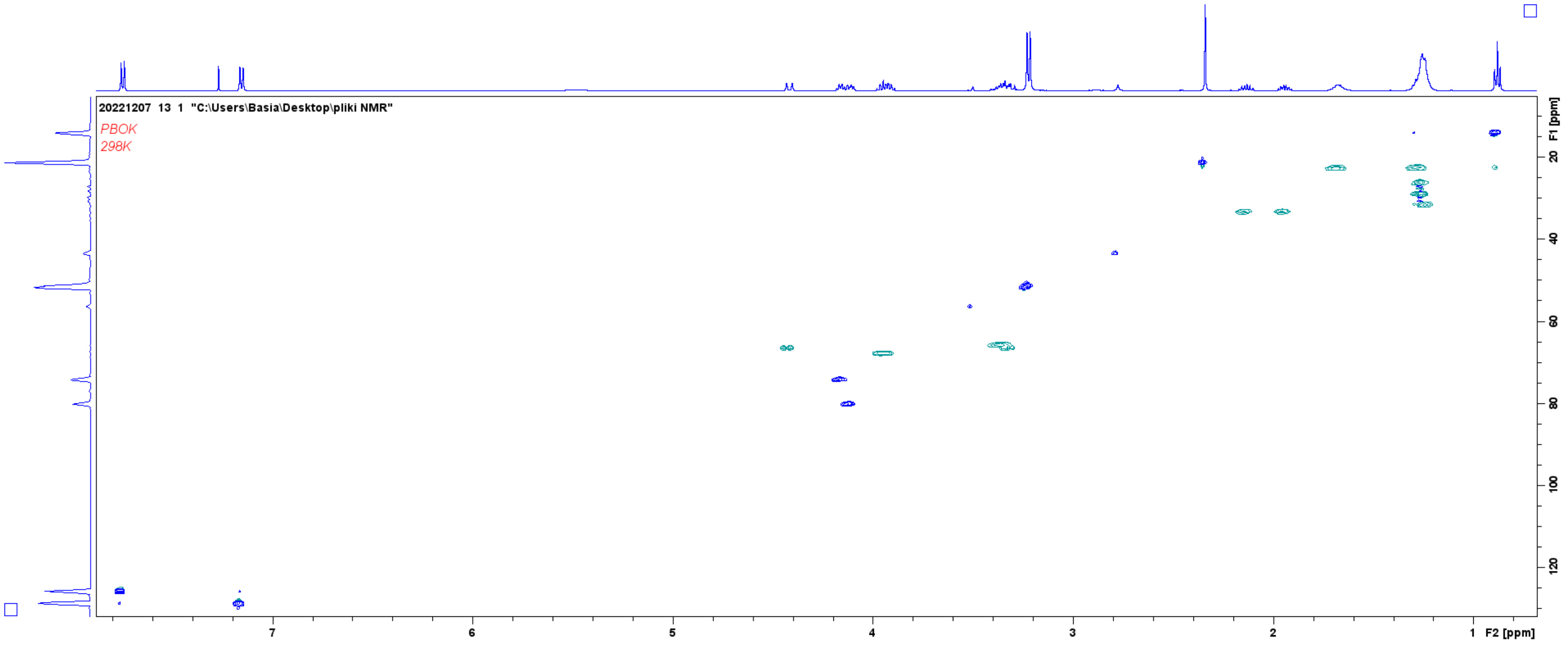

Figure S20. HSQC spectrum of *N*-[(2*R*,3*S*)-(3-hydroxyoxolan-2-yl)methyl]-*N*,*N*-dimethyl-*N*-octylammonium tosylate (**5c**).

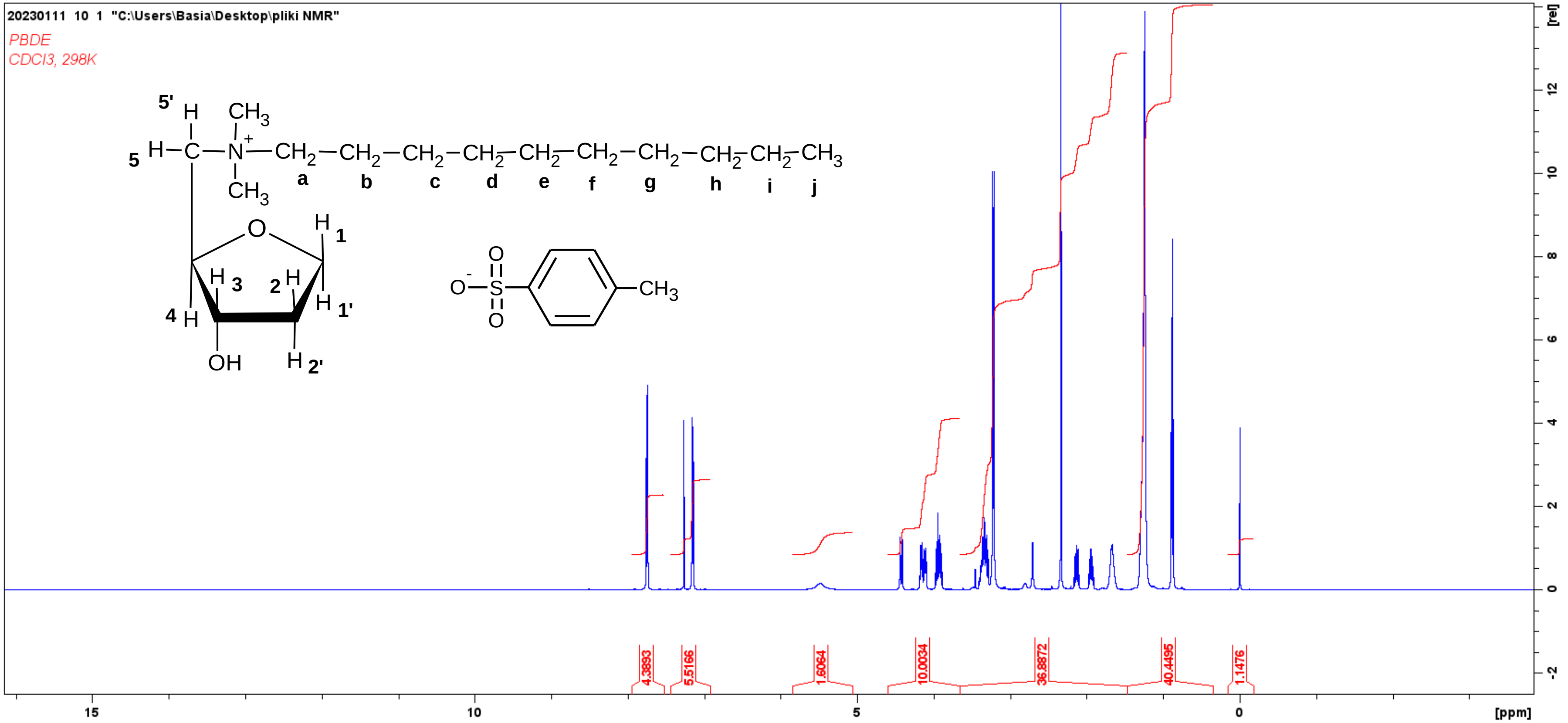

Figure S21. ^{1}H NMR spectrum (500 MHz, ~~D_2O~~ $CDCl_3$) of *N*-[(2*R*,3*S*)-(3-hydroxyoxolan-2-yl)methyl]- *N*-decyl-*N*,*N*-dimethylammonium tosylate (**5d**).

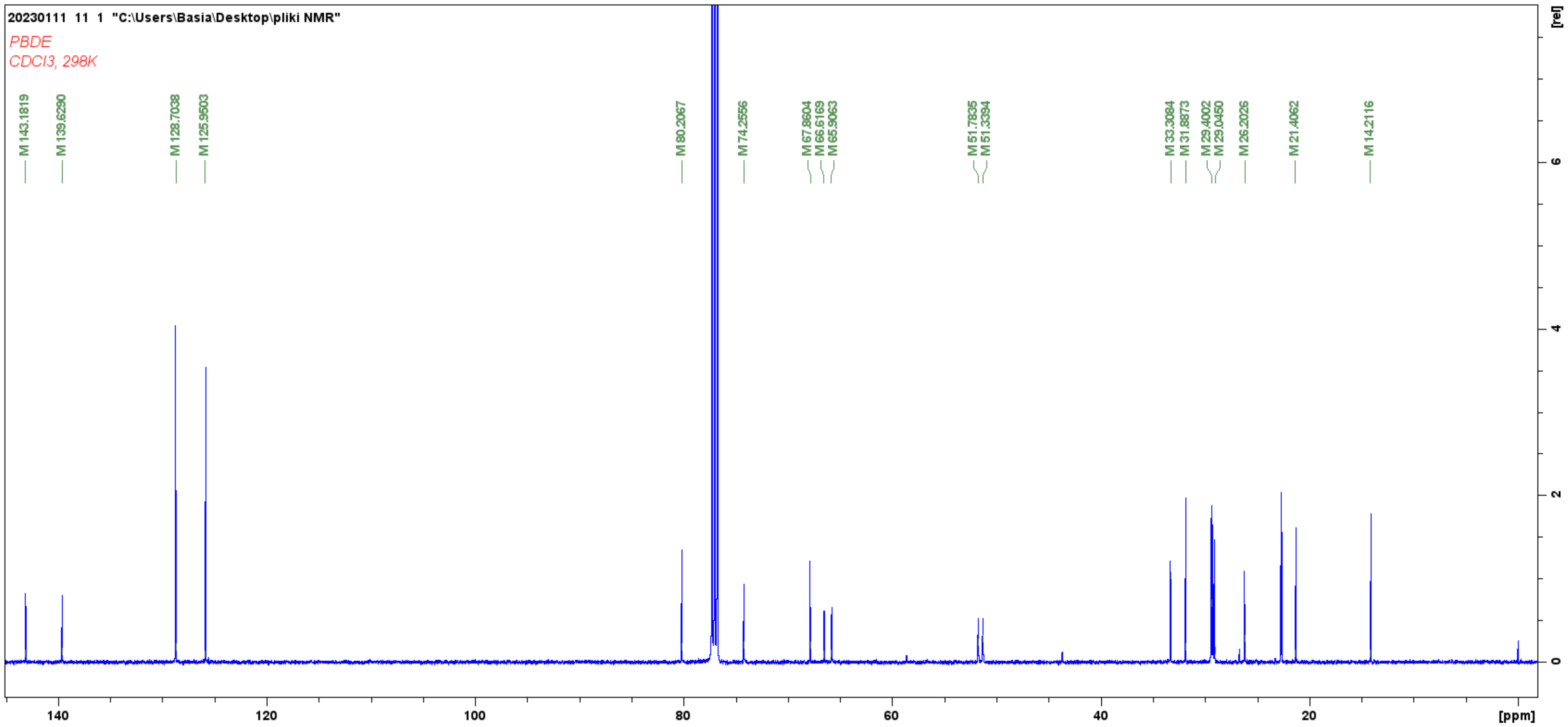

Figure S22. ^{13}C NMR spectrum (125 MHz, ~~D_2O~~ $CDCl_3$) of *N*-[(2*R*,3*S*)-(3-hydroxyoxolan-2-yl)methyl]- *N*-decyl-*N*,*N*-dimethylammonium tosylate (**5d**).

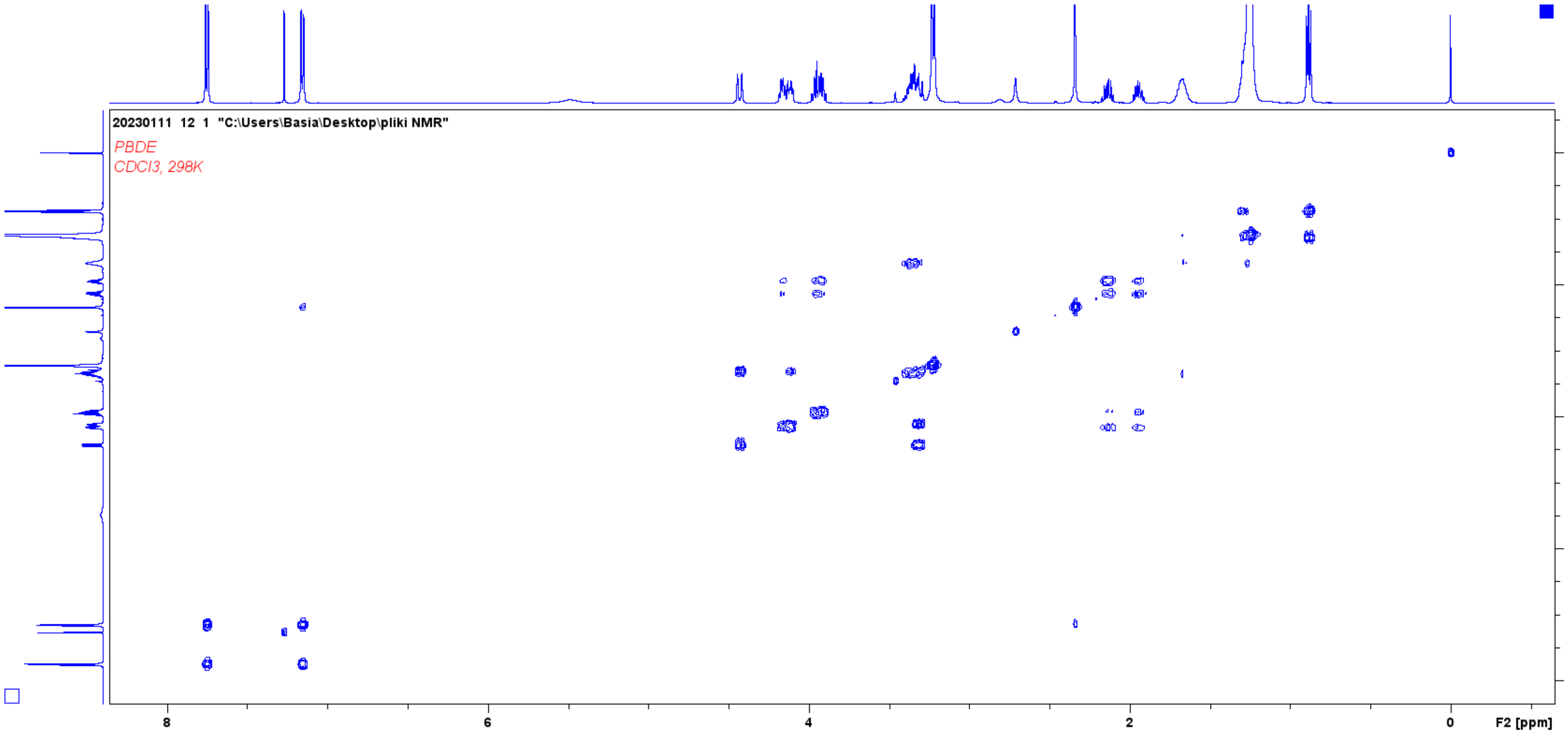

Figure S23. COSY spectrum of *N*-[(2*R*,3*S*)-(3-hydroxyoxolan-2-yl)methyl]- *N*-decyl-*N*,*N*-dimethylammonium tosylate (**5d**).

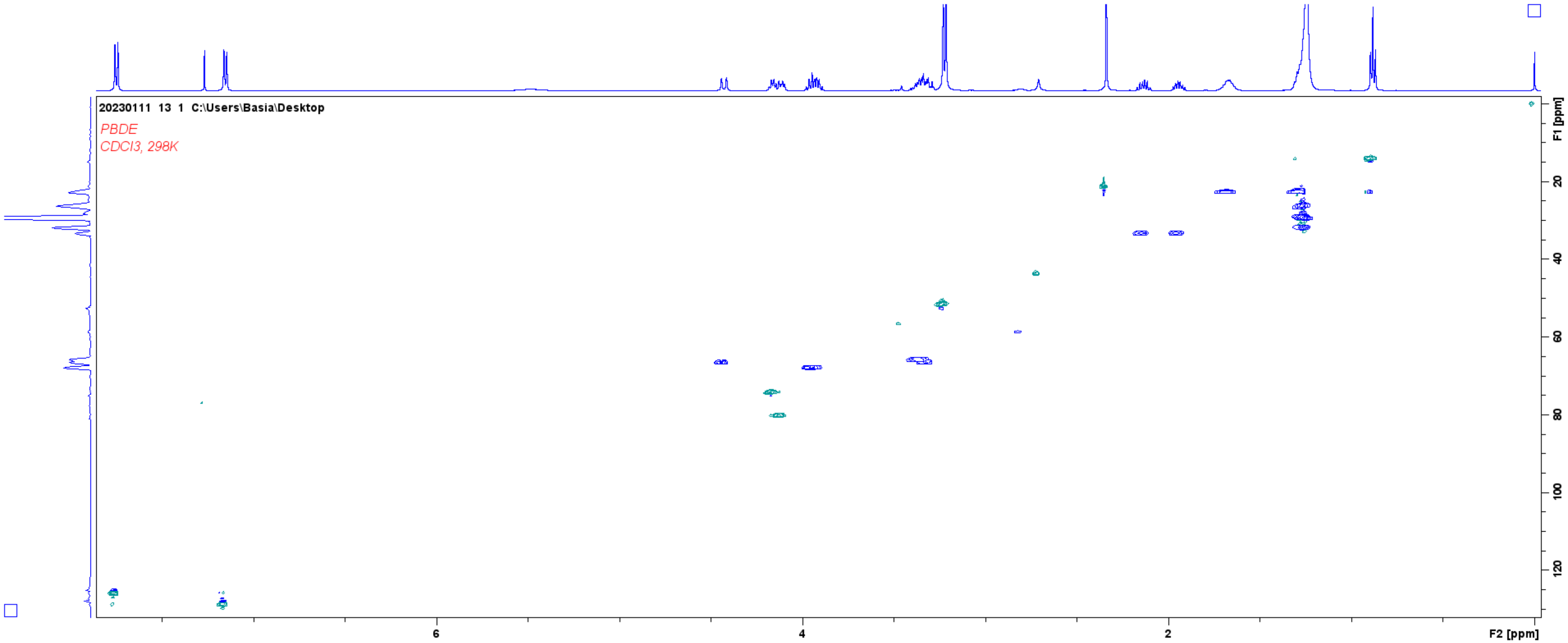

Figure S24. HSQC spectrum of *N*-[(2*R*,3*S*)-(3-hydroxyoxolan-2-yl)methyl]- *N*-decyl-*N*,*N*-dimethylammonium tosylate (**5d**).

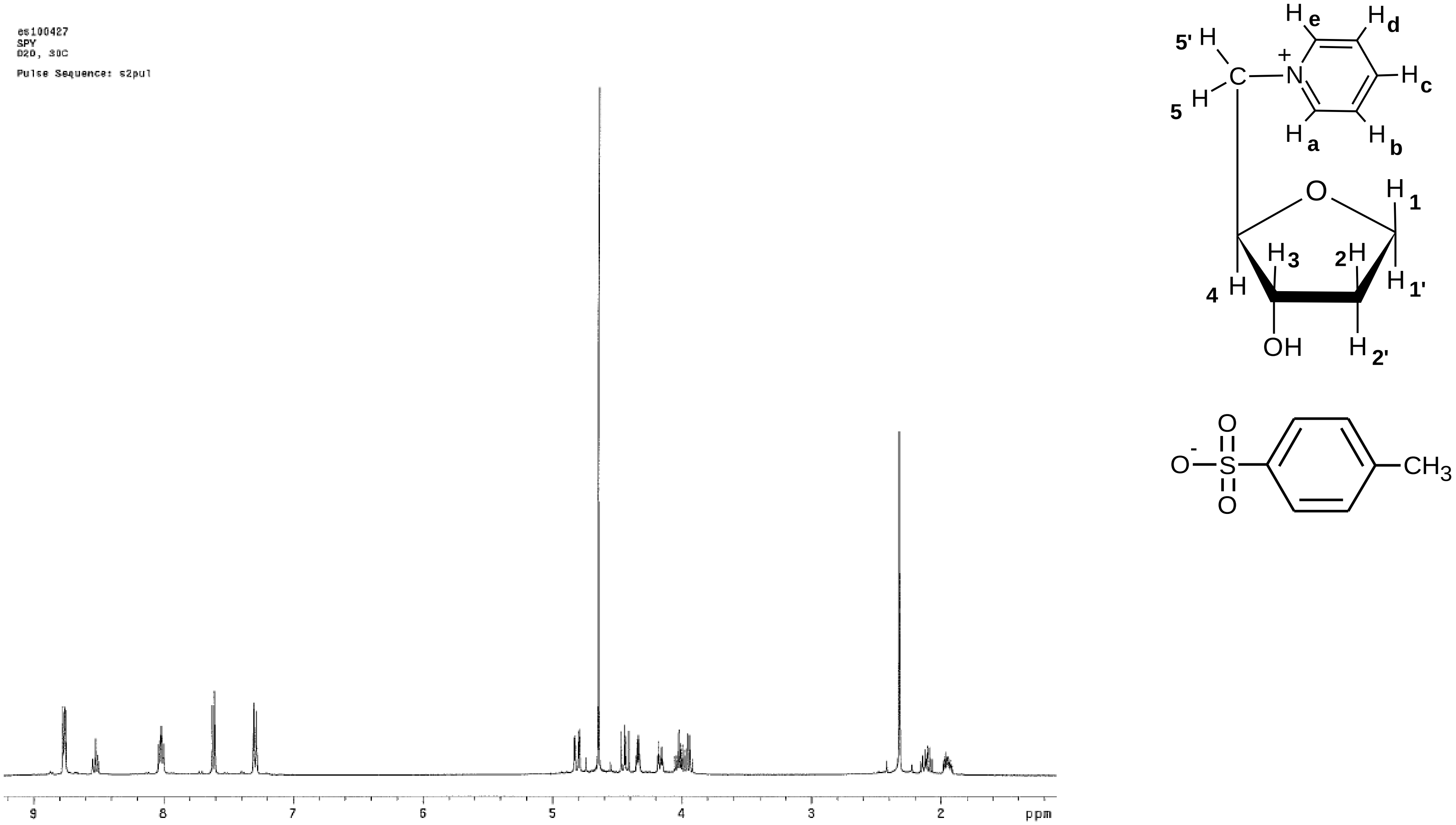

Figure S25. ^{1}H NMR spectrum (400 MHz, D_2O) of *N*-[(2*R*,3*S*)-(3-hydroxyoxolan-2-yl)methyl]pyridinium tosylate (**5e**).

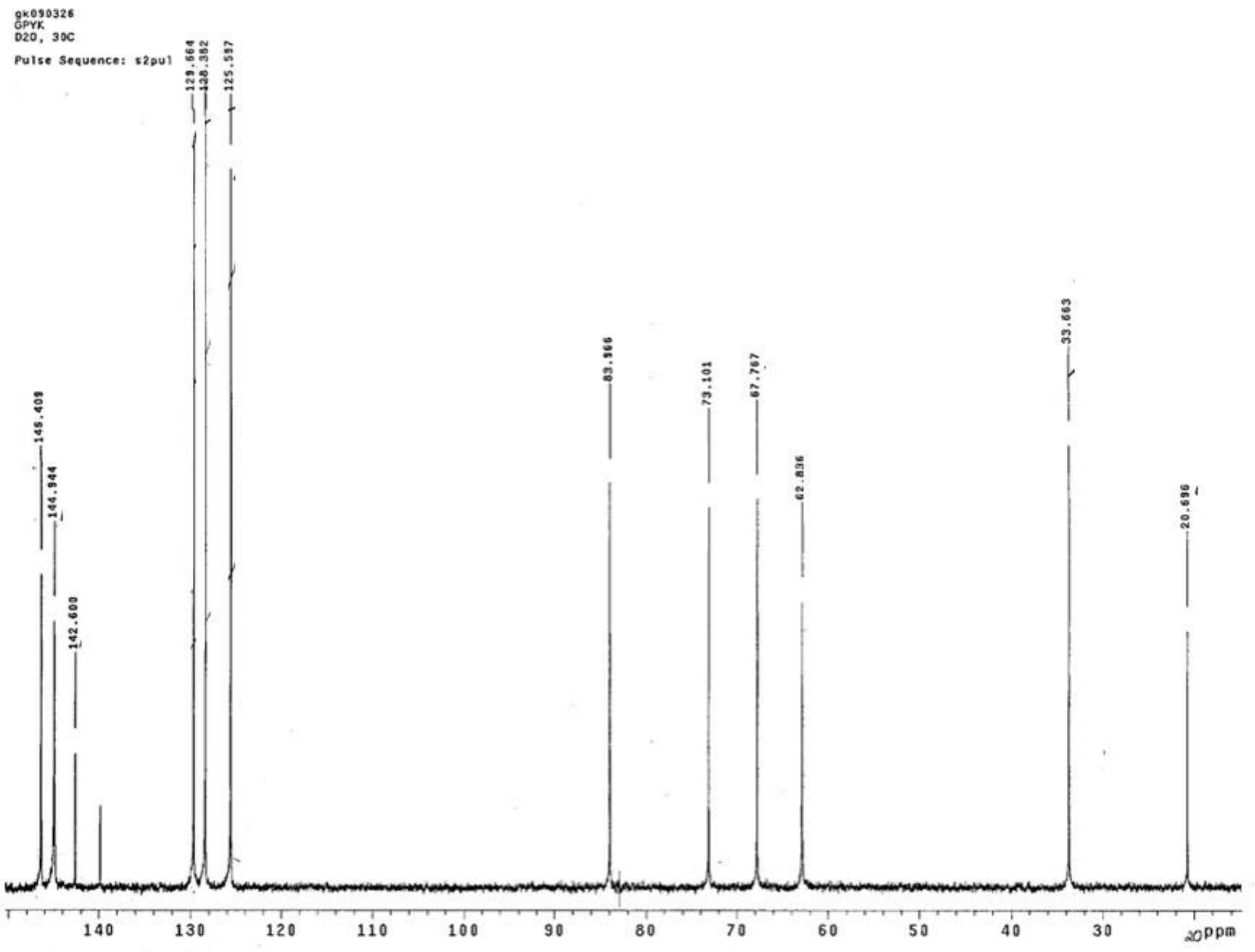

Figure S26. ^{13}C NMR spectrum (100 MHz, D_2O) of *N*-[(2*R*,3*S*)-(3-hydroxyoxolan-2-yl)methyl]pyridinium tosylate (**5e**).

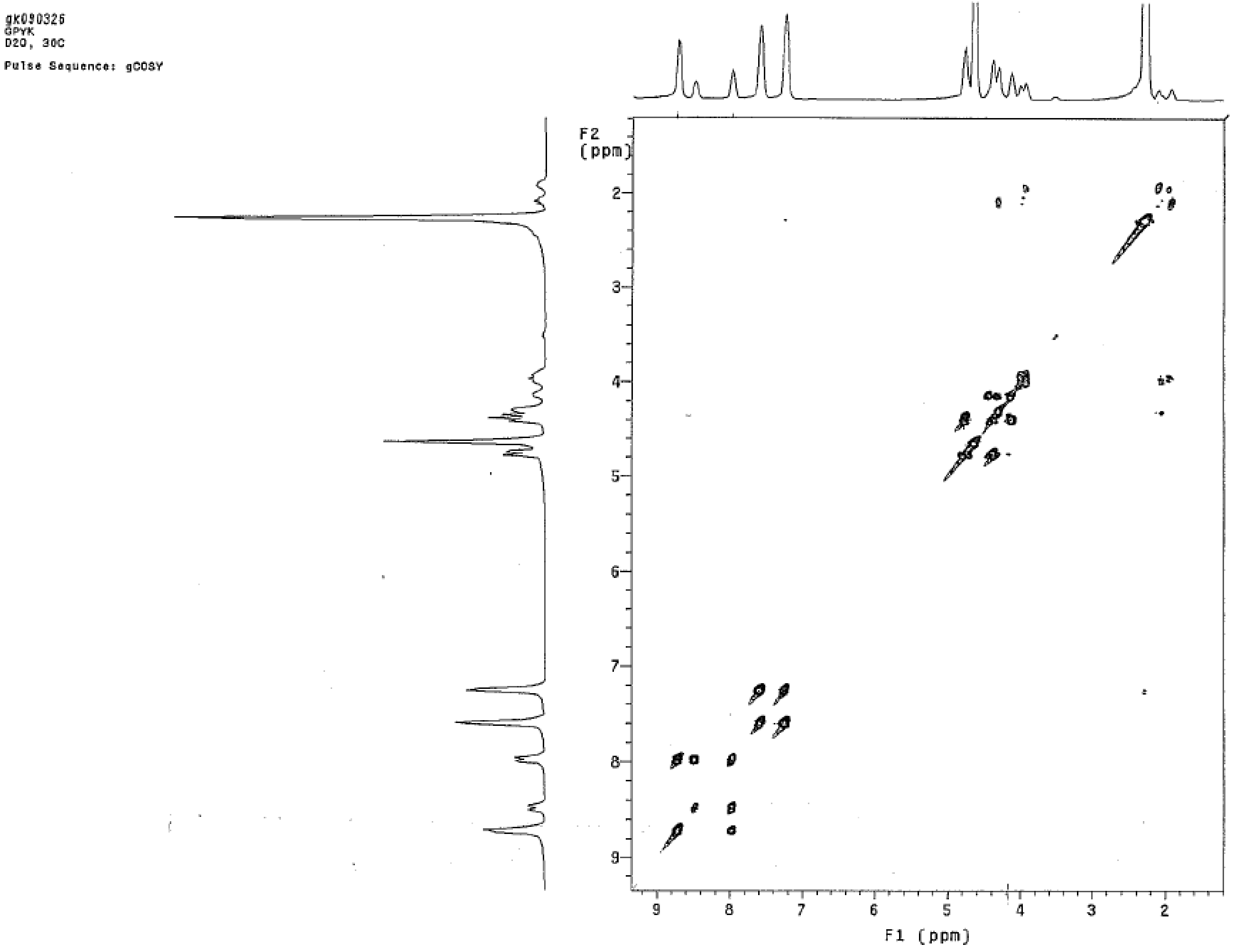

Figure S27. COSY spectrum of *N*-[(2*R*,3*S*)-(3-hydroxyoxolan-2-yl)methyl]pyridinium tosylate (**5e**).

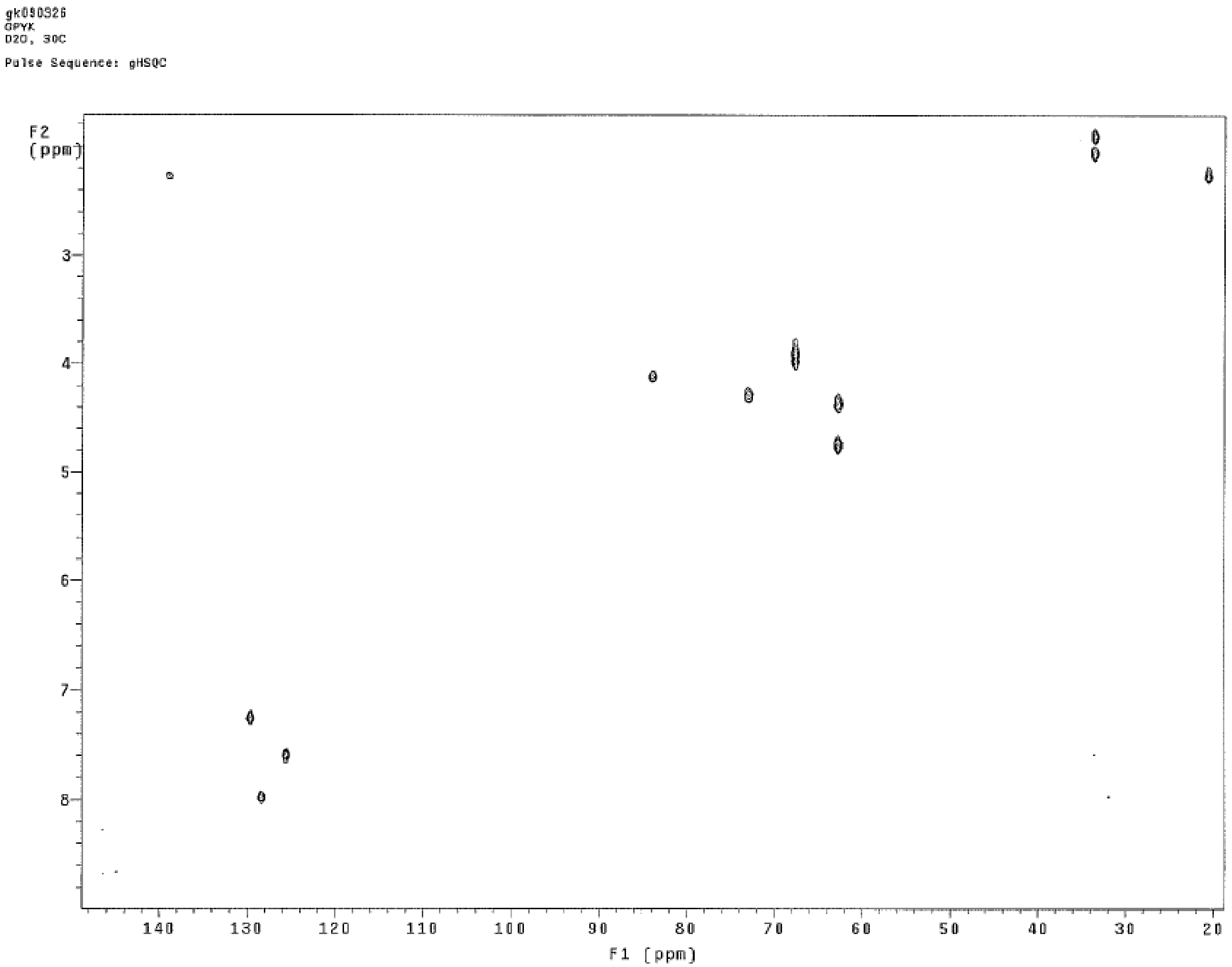

Figure S28. HSQC spectrum of *N*-[(2*R*,3*S*)-(3-hydroxyoxolan-2-yl)methyl]pyridinium tosylate (**5e**).

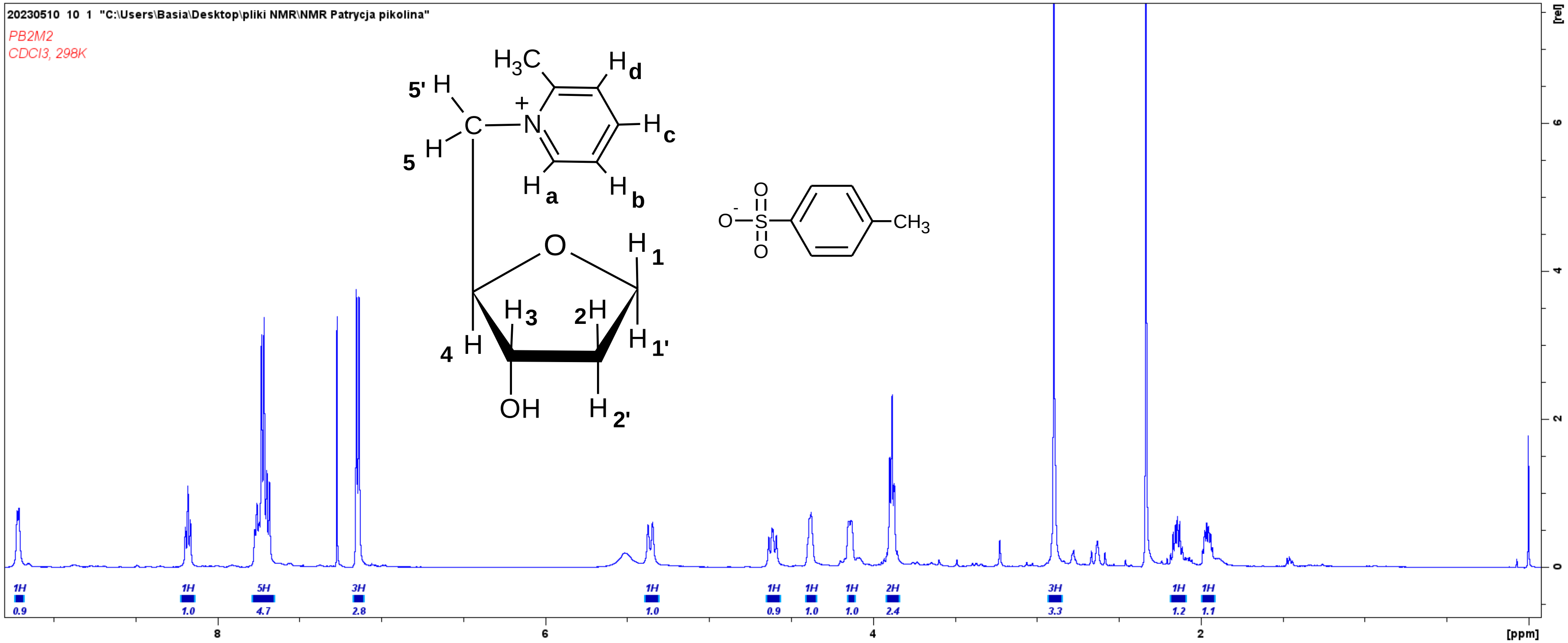

Figure S29. ^{1}H NMR spectrum (500 MHz, ~~D_2O~~ $CDCl_3$) of *N*-[(2*R*,3*S*)-(3-hydroxyoxolan-2-yl)methyl]-2-methylpyridinium tosylate (**5f**).

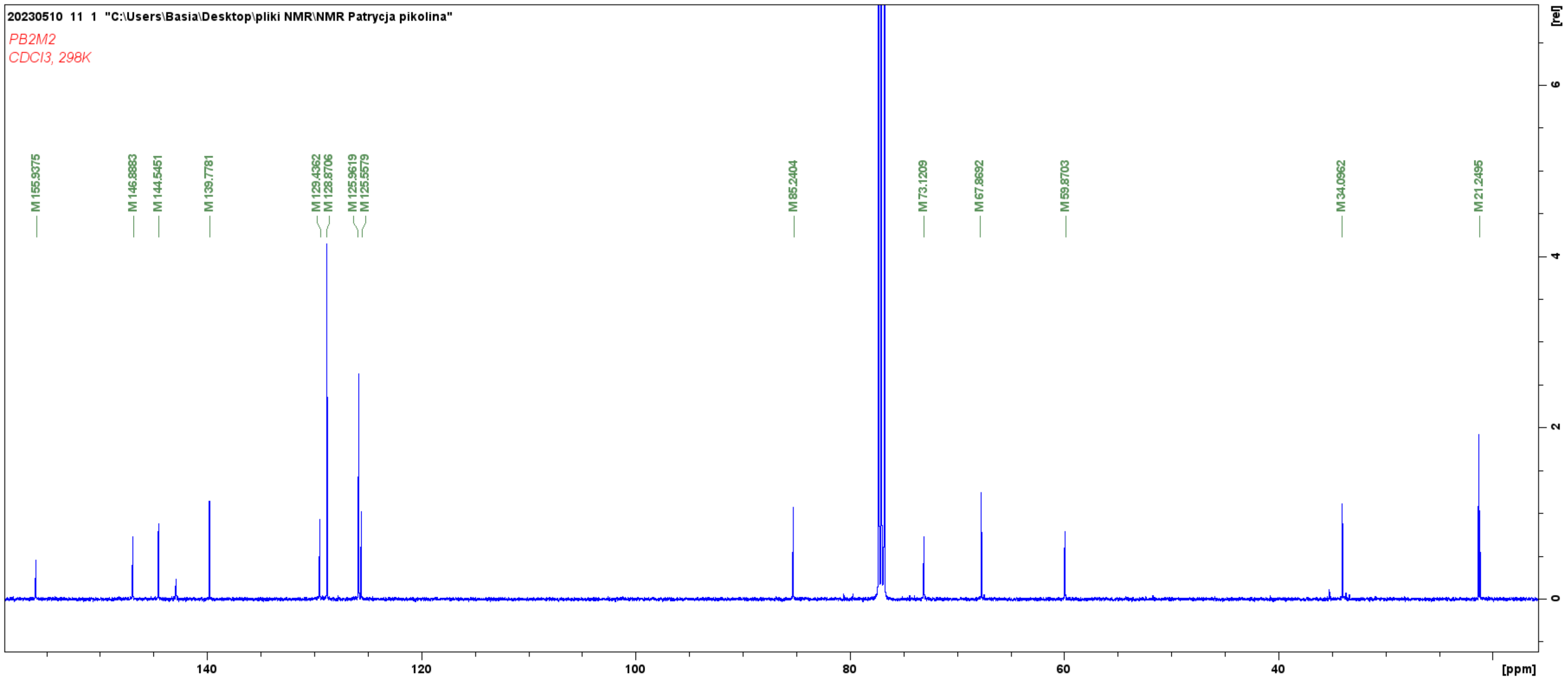

Figure S30. ^{13}C NMR spectrum (125 MHz, ~~D_2O~~ $CDCl_3$) of *N*-[(2*R*,3*S*)-(3-hydroxyoxolan-2-yl)methyl]-2-methylpyridinium tosylate (**5f**).

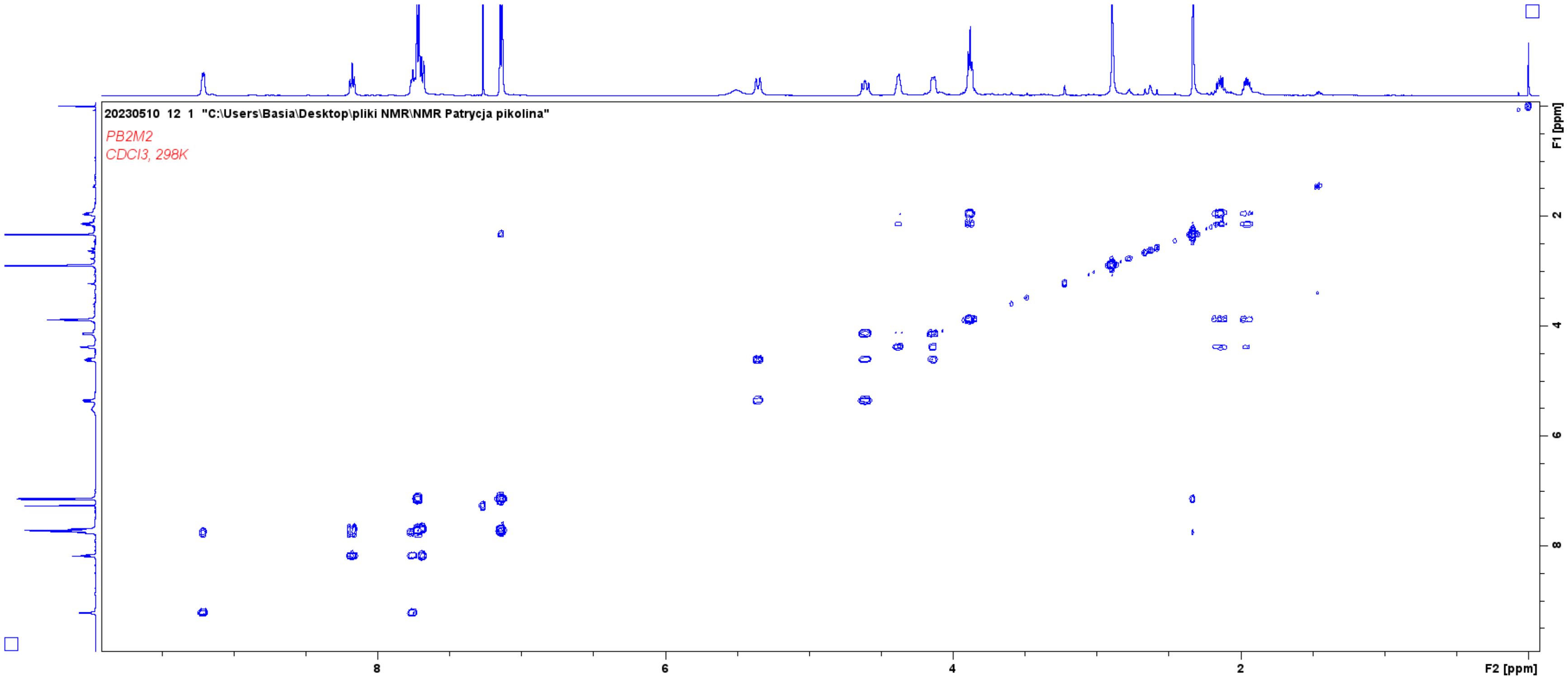

Figure S31. COSY spectrum of *N*-[(2*R*,3*S*)-(3-hydroxyoxolan-2-yl)methyl]-2-methylpyridinium tosylate (**5f**).

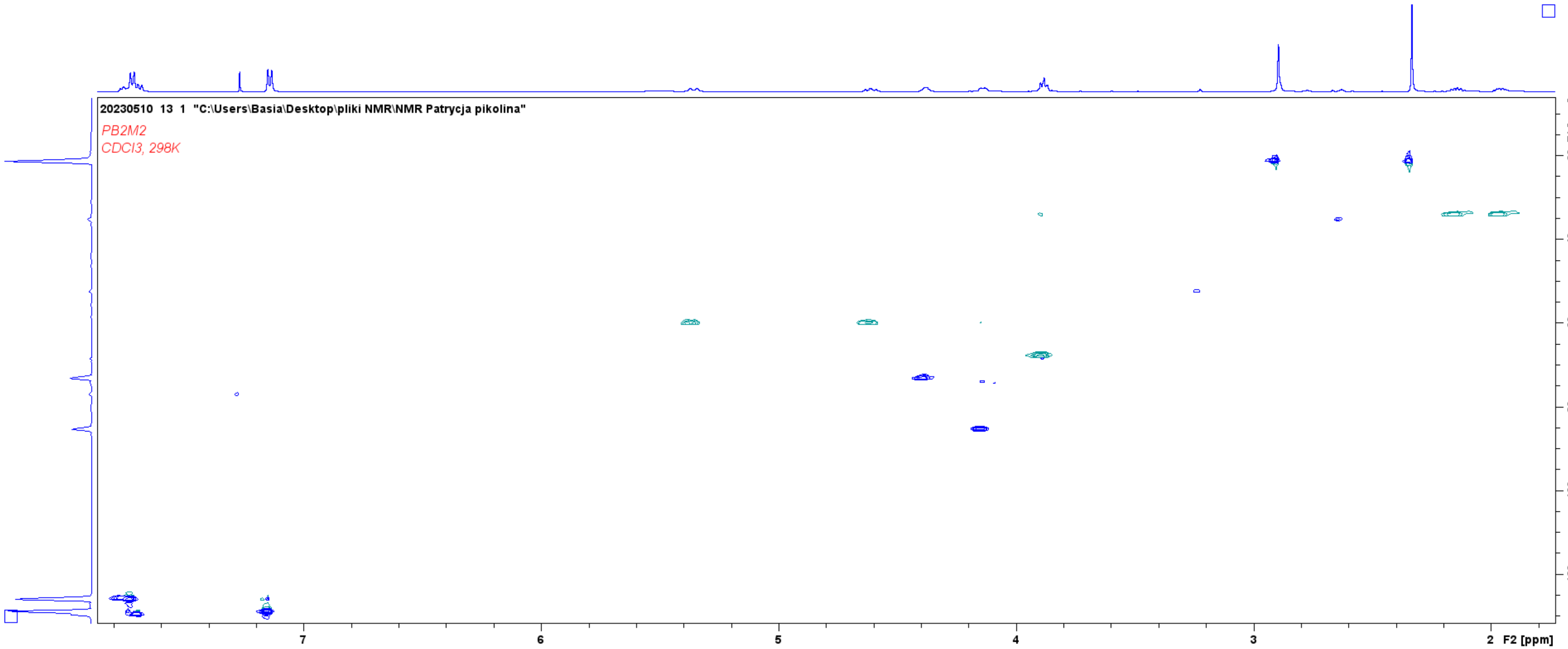

Figure S32. HSQC spectrum of *N*-[(2*R*,3*S*)-(3-hydroxyoxolan-2-yl)methyl]-2-methylpyridinium tosylate (**5f**).

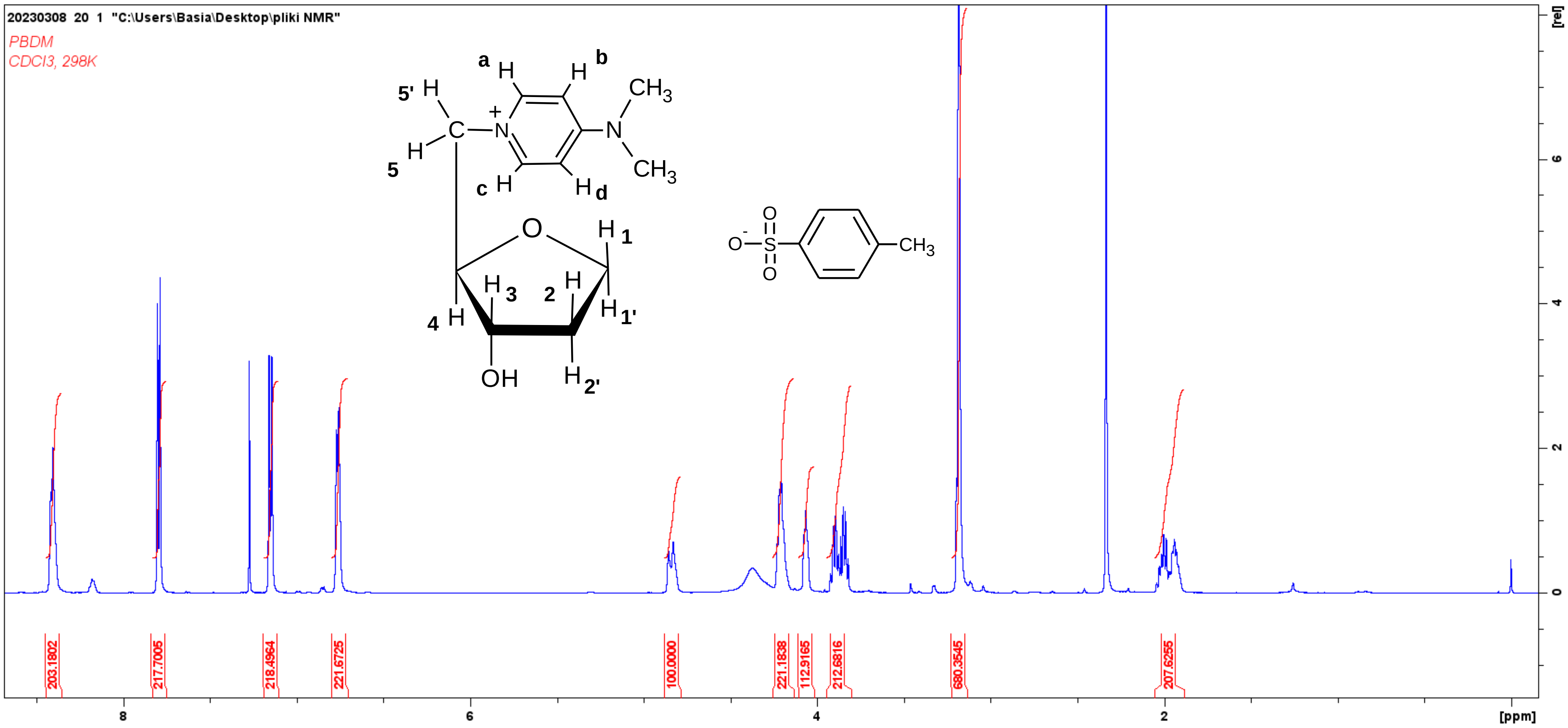

Figure S33. ^{1}H NMR spectrum (500 MHz, ~~D_2O~~ $CDCl_3$) of *N*-[(2*R*,3*S*)-(3-hydroxyoxolan-2-yl)methyl]-4-(*N*,*N*-dimethylamino)pyridinium tosylate (**5g**).

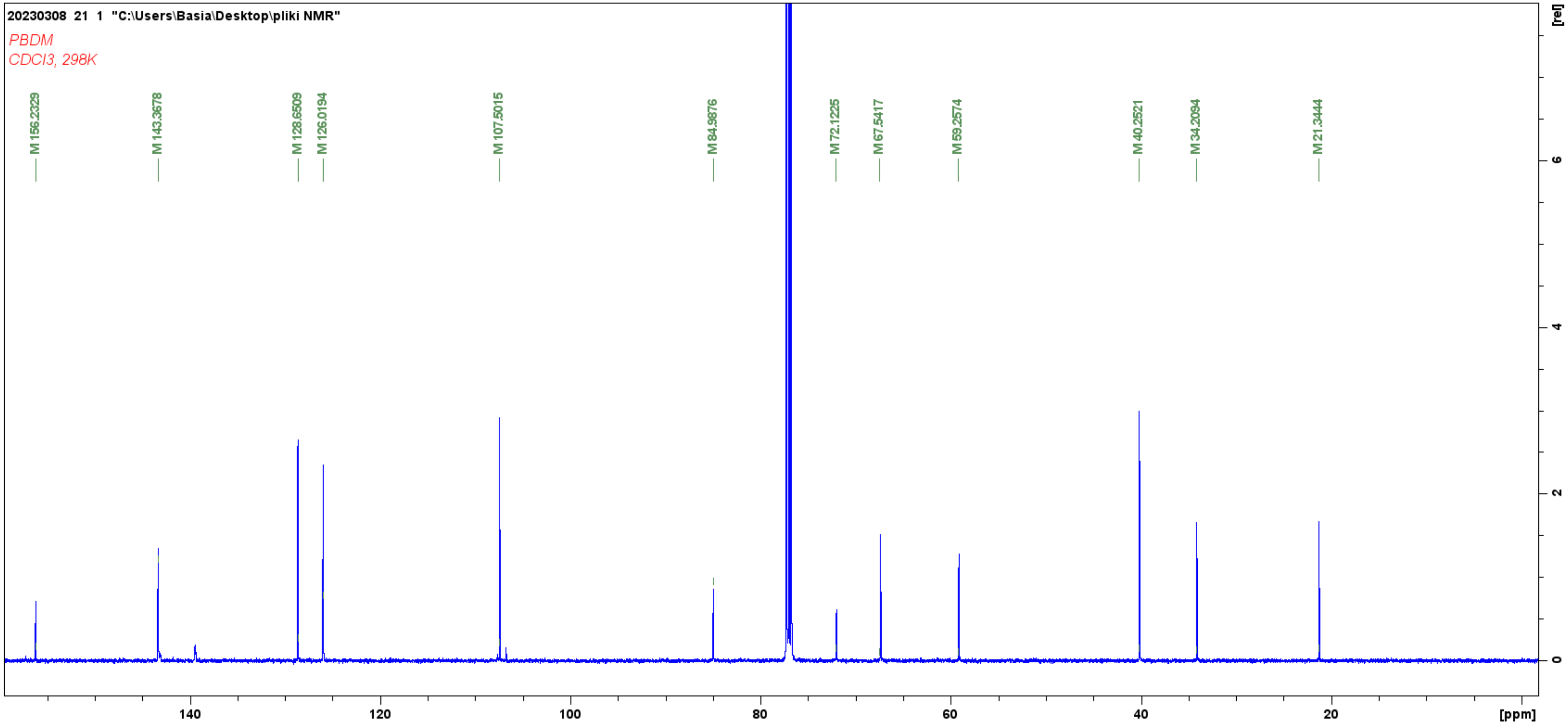

Figure S34. ^{13}C NMR spectrum (125 MHz, ~~D_2O~~ $CDCl_3$) of *N*-[(2*R*,3*S*)-(3-hydroxyoxolan-2-yl)methyl]-4-(*N*,*N*-dimethylamino)pyridinium tosylate (**5g**).

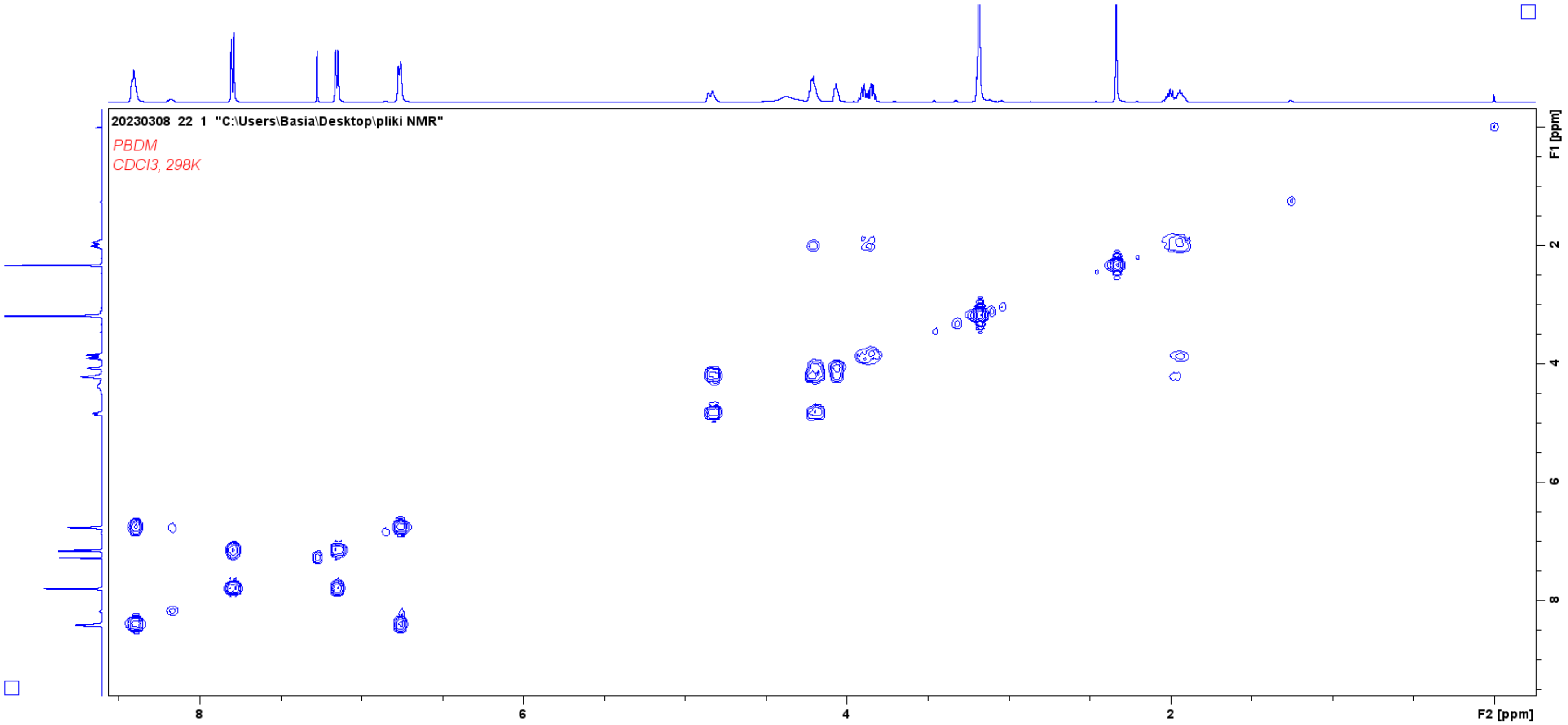

Figure S35. COSY spectrum of *N*-[(2*R*,3*S*)-(3-hydroxyoxolan-2-yl)methyl]-4-(*N*,*N*-dimethylamino)pyridinium tosylate (**5g**).

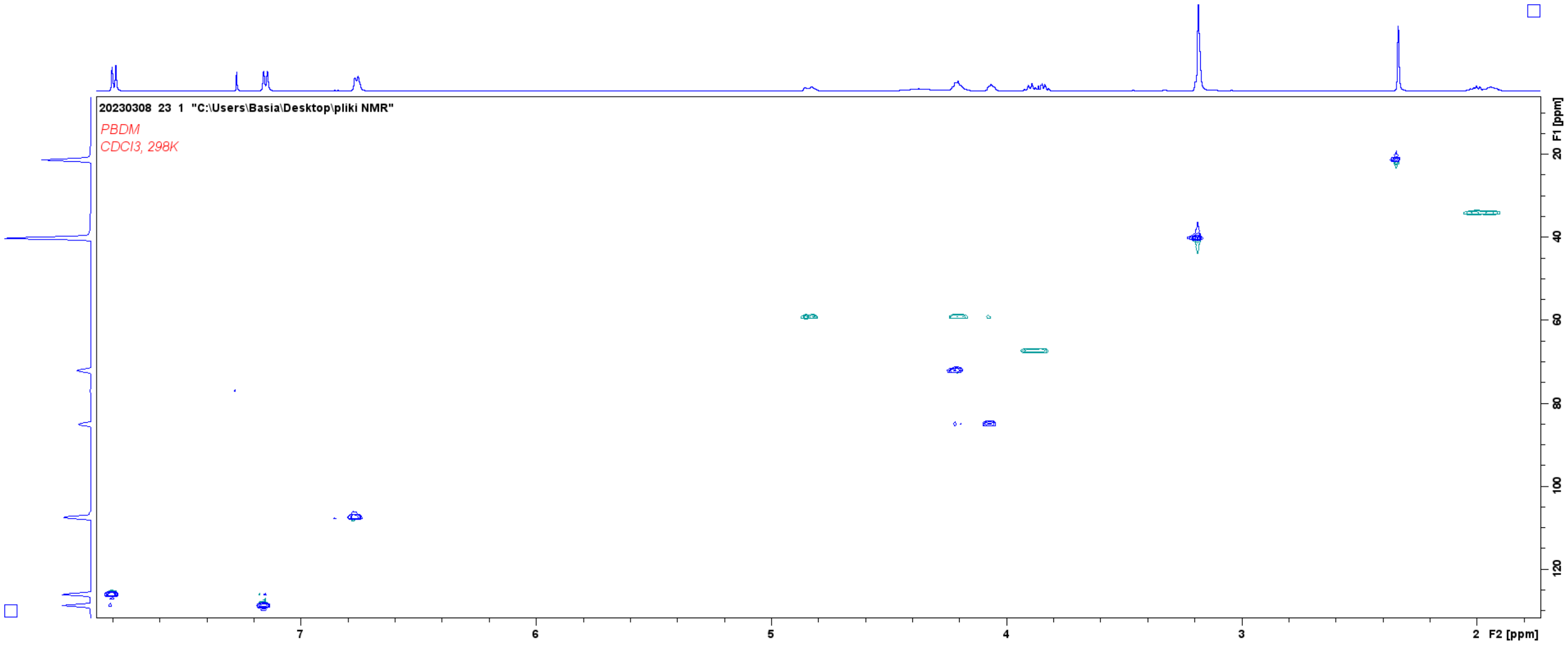

Figure S36. HSQC spectrum of *N*-[(2*R*,3*S*)-(3-hydroxyoxolan-2-yl)methyl]-4-(*N*,*N*-dimethylamino)pyridinium tosylate (**5g**).

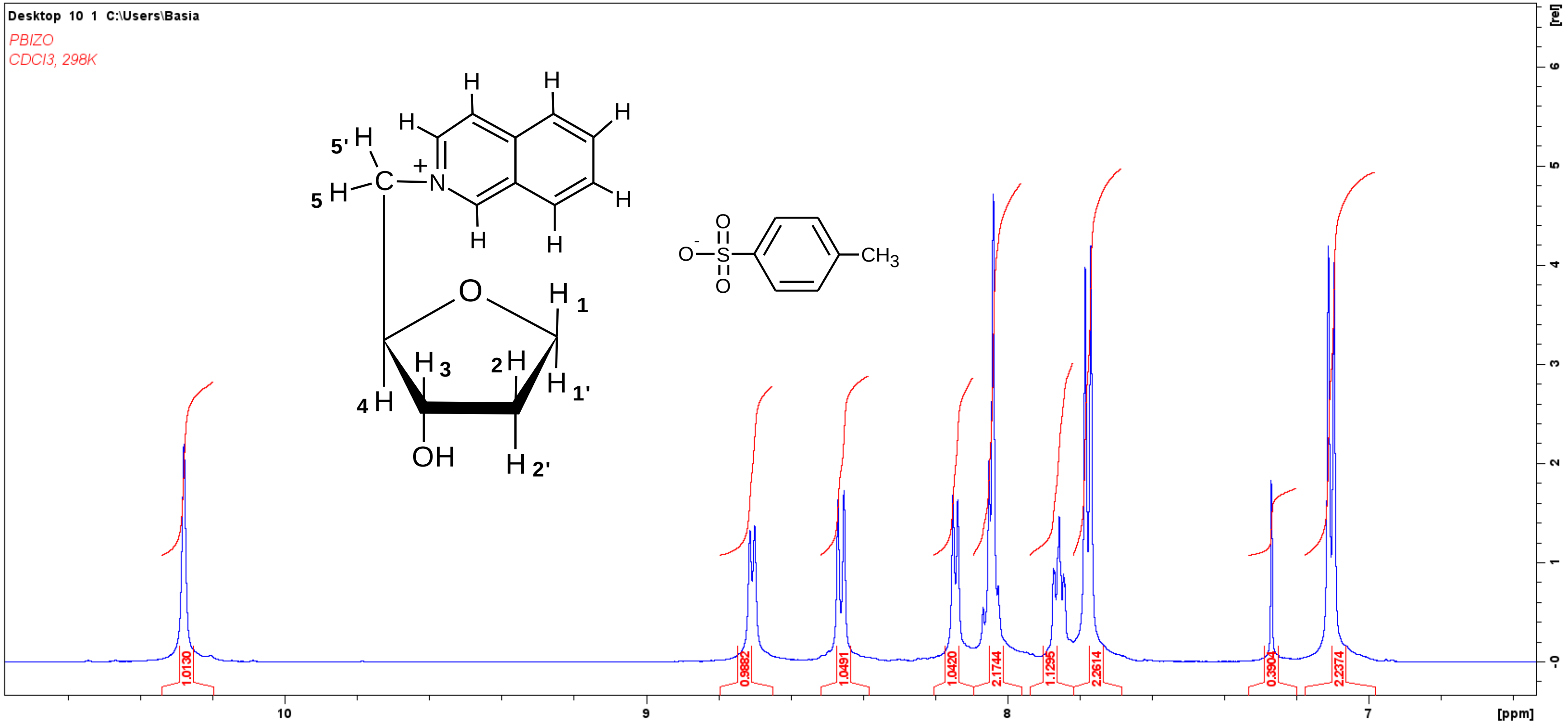

Figure S37. ^{1}H NMR spectrum (500 MHz, ~~D_2O~~ $CDCl_3$) of *N*-[(2*R*,3*S*)-(3-hydroxyoxolan-2-yl)methyl]isoquinolinium tosylate (**5h**).

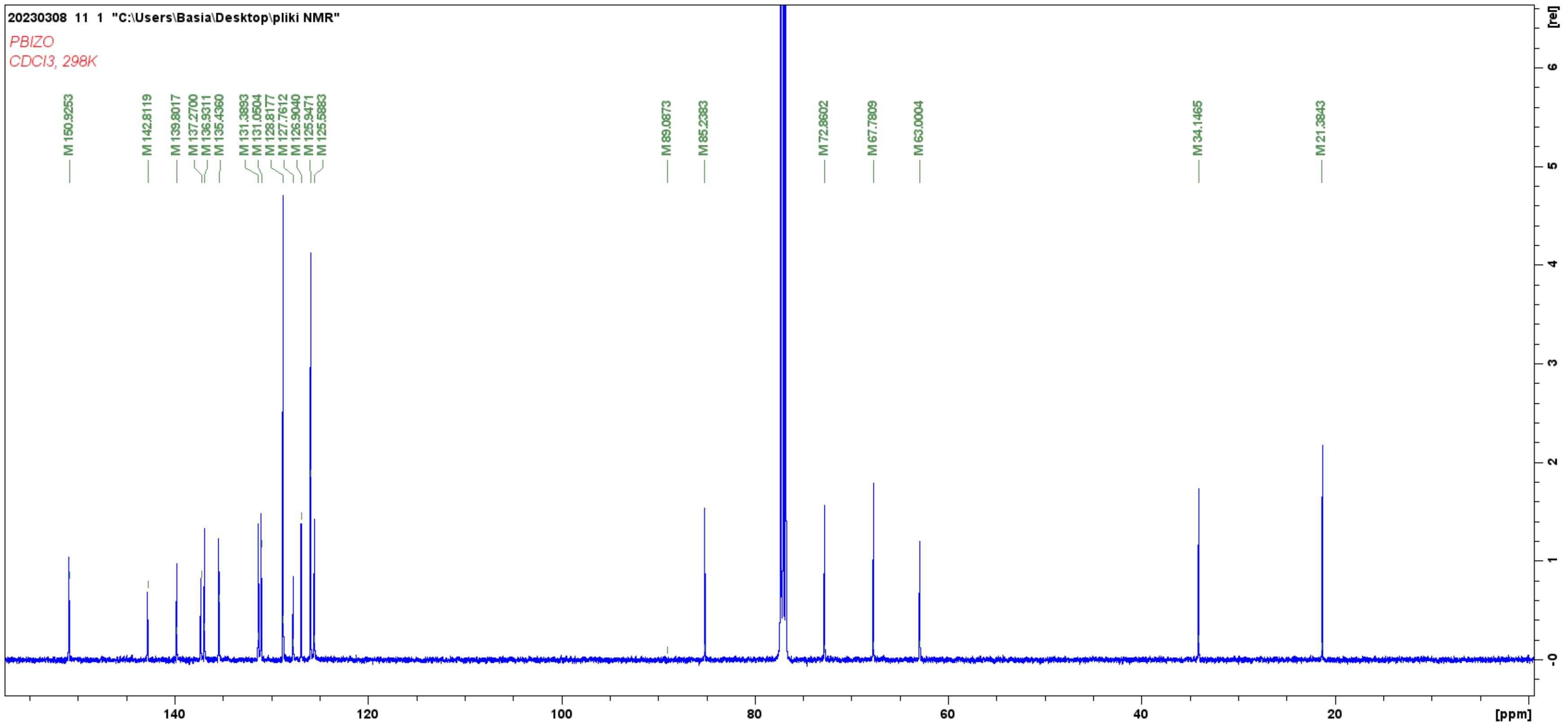

Figure S38. ^{13}C NMR spectrum (125 MHz, ~~D_2O~~ $CDCl_3$) of *N*-[(2*R*,3*S*)-(3-hydroxyoxolan-2-yl)methyl]isoquinolinium tosylate (**5h**).

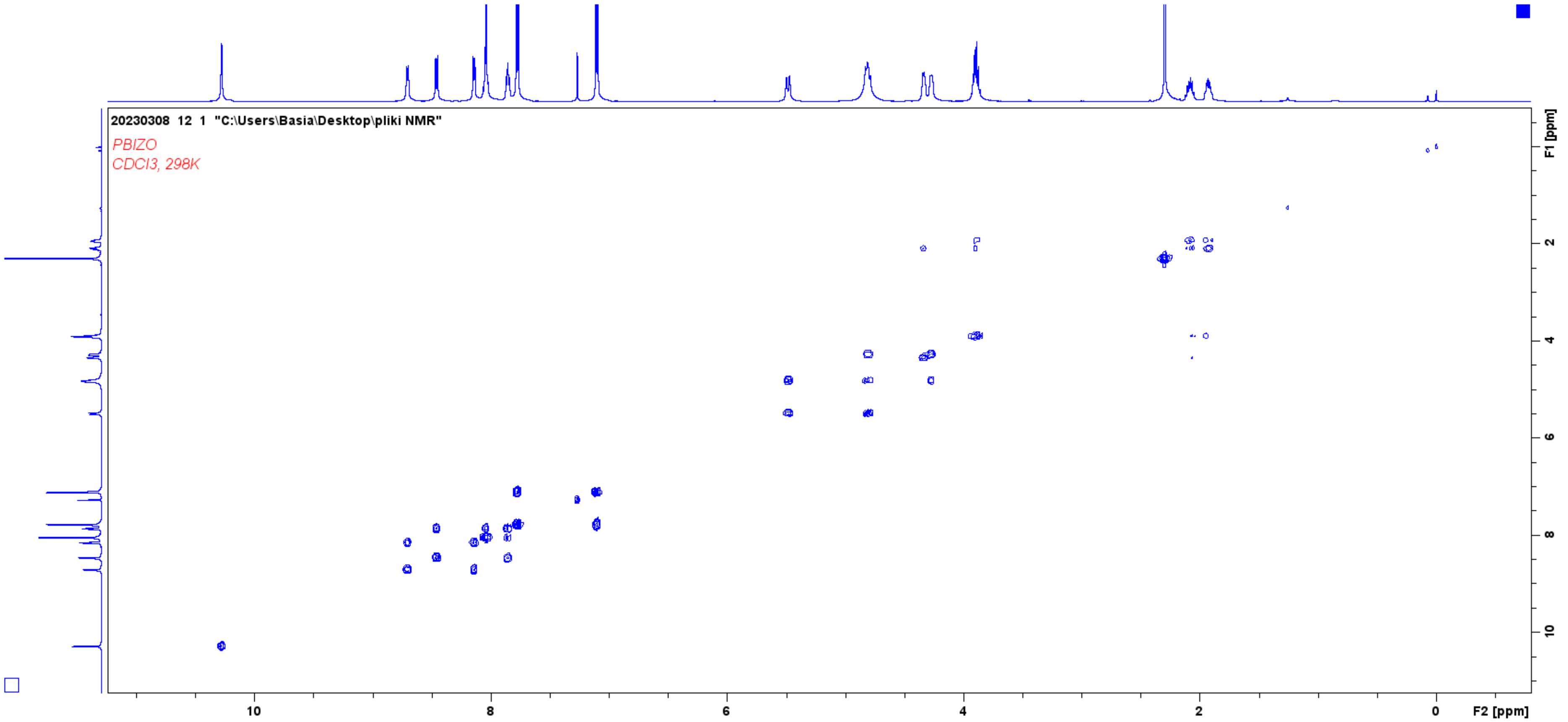

Figure S39. COSY spectrum of *N*-[(2*R*,3*S*)-(3-hydroxyoxolan-2-yl)methyl]isoquinolinium tosylate (**5h**).

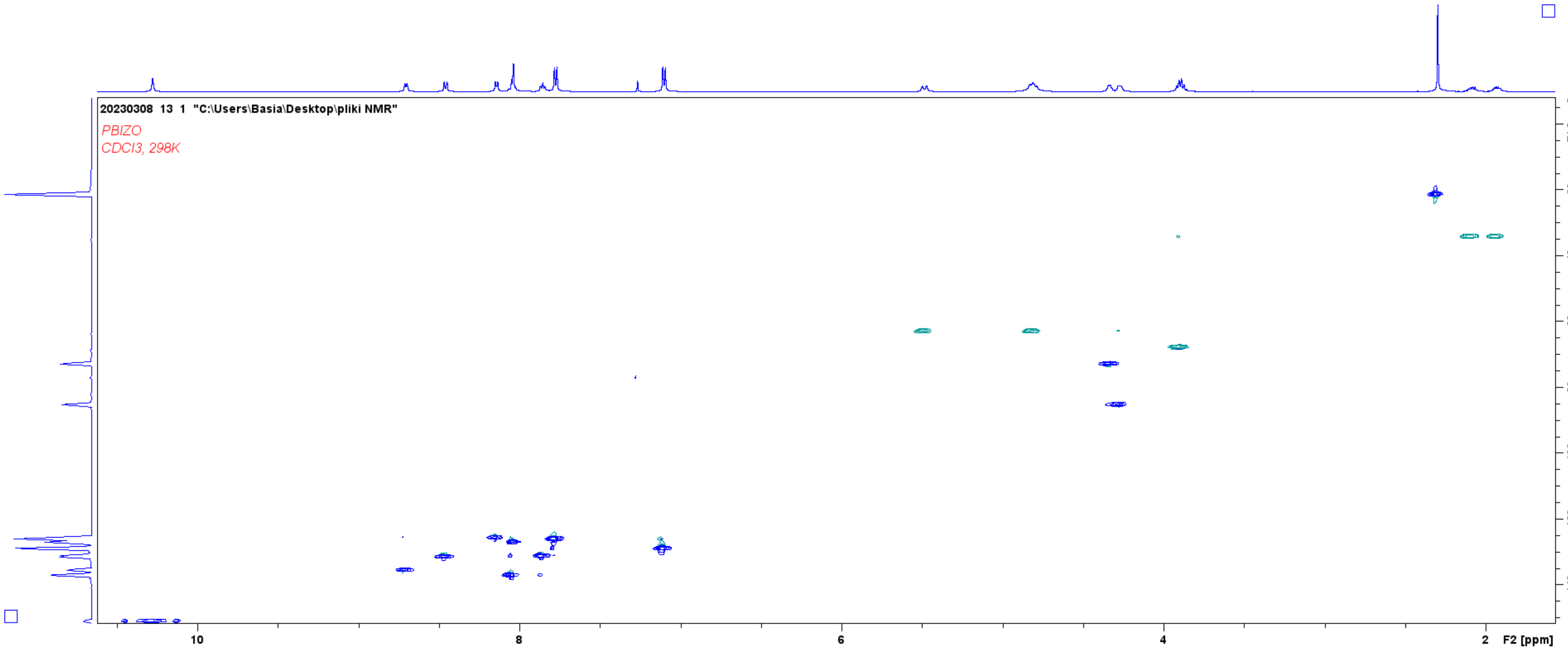

Figure S40. HSQC spectrum of *N*-[(2*R*,3*S*)-(3-hydroxyoxolan-2-yl)methyl]isoquinolinium tosylate (**5h**).

MALDITOF MS SPECTRA

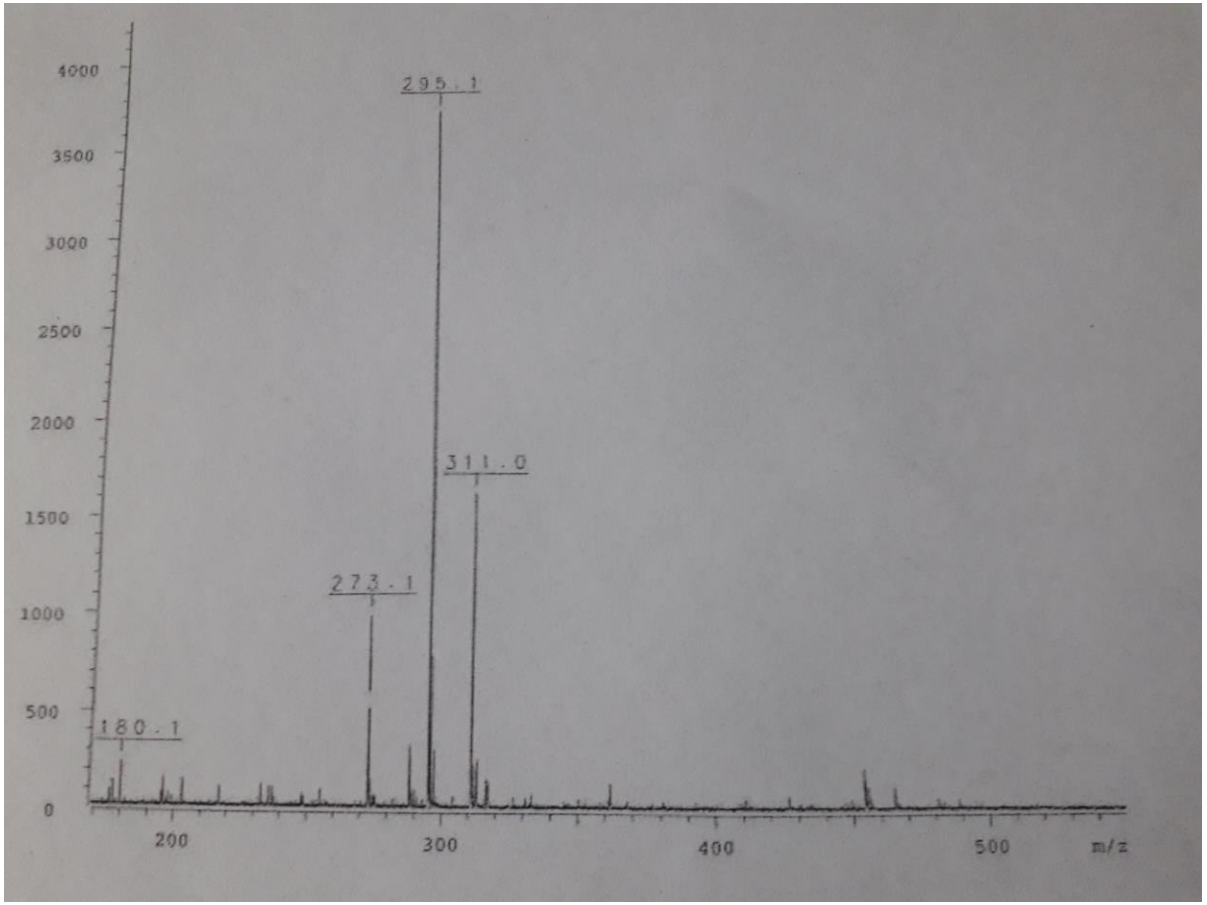

Figure S41. MALDITOF MS spectrum of (2*R*,3*S*)-2-(*O*-tosylmethoxyl)oxolan-3-ol (**4**), m/z 273.10 ($[M+H]^+$), 295.1 ($[M+Na]^+$), 311.0 ($[M+K]^+$).

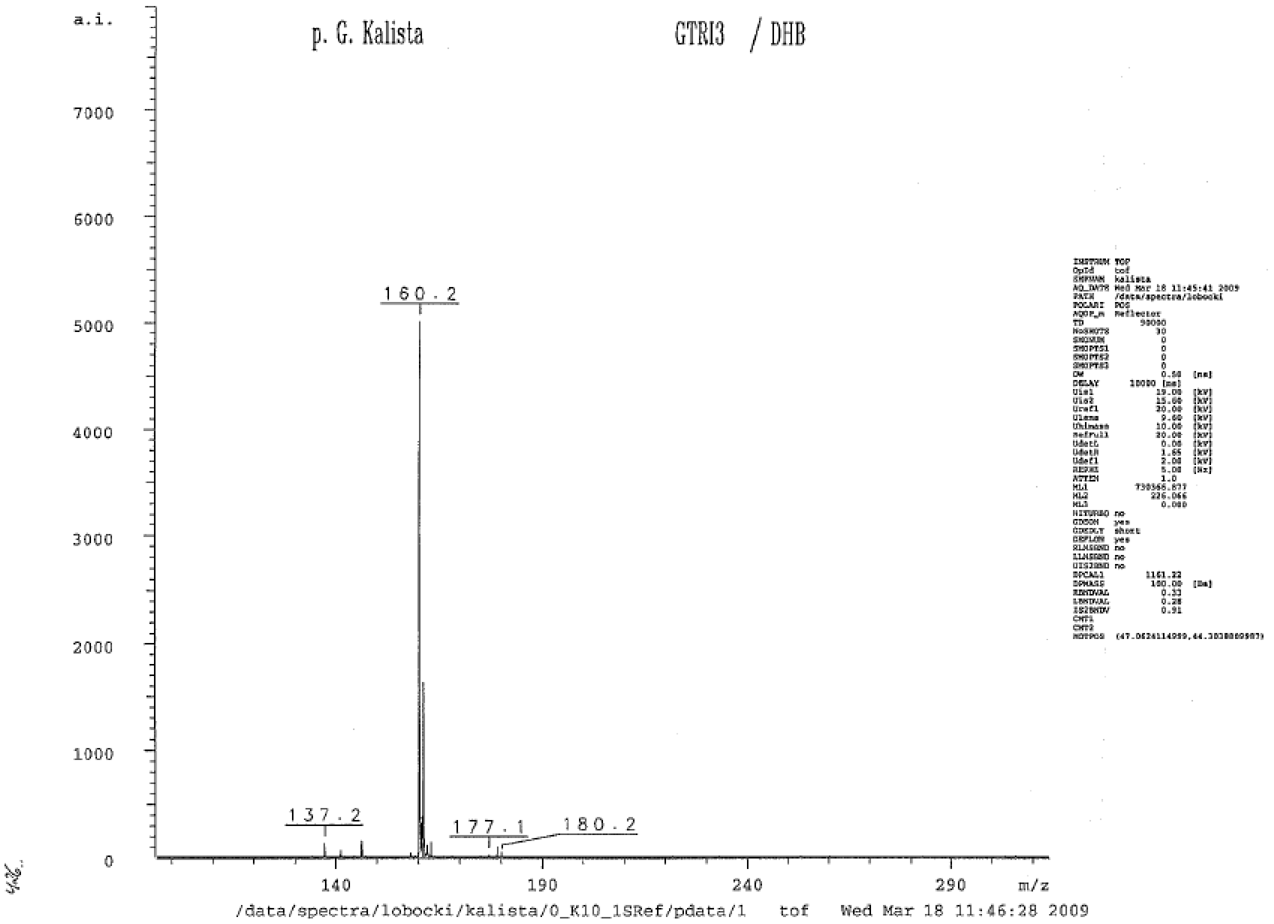

Figure S42. MALDITOF MS spectrum of *N*-[(2*R*,3*S*)-(3-hydroxyoxolan-2-yl)methyl]-*N*,*N*,*N*-trimethylamonium tosylate (**5a**), m/z 160,20 ([M-OTs]$^{+}$).

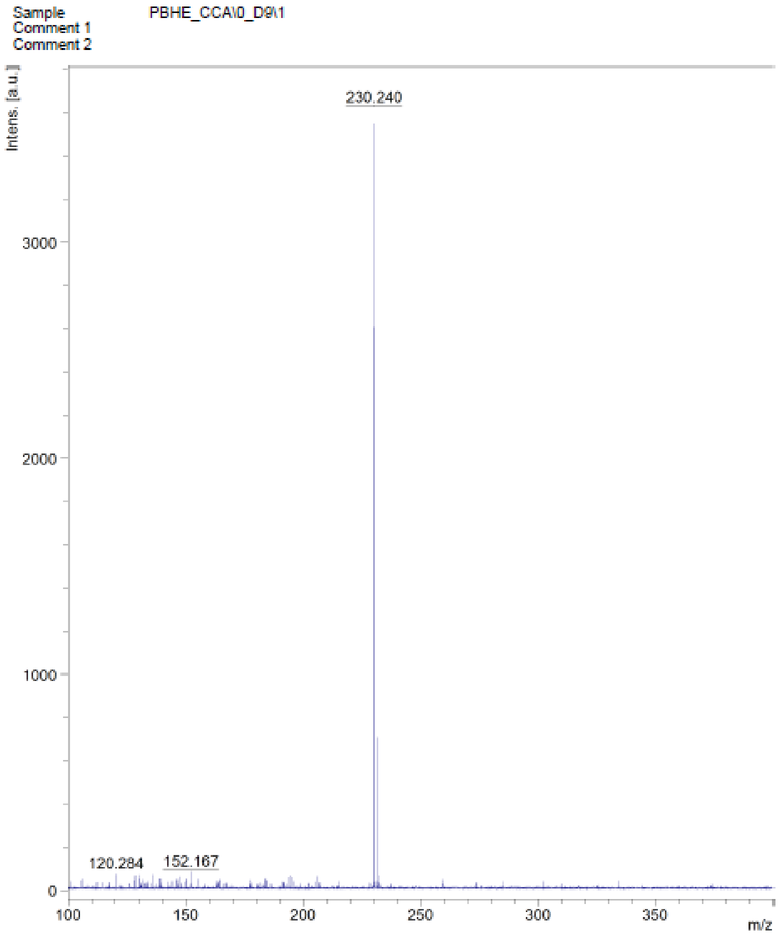

Figure S43. MALDITOF MS spectrum of *N*-[(2*R*,3*S*)-(3-hydroxyoxolan-2-yl)methyl]-*N*-hexyl-*N*,*N*-dimethylammonium tosylate (**5b**), m/z 230,240 ([M-OTs]$^+$).

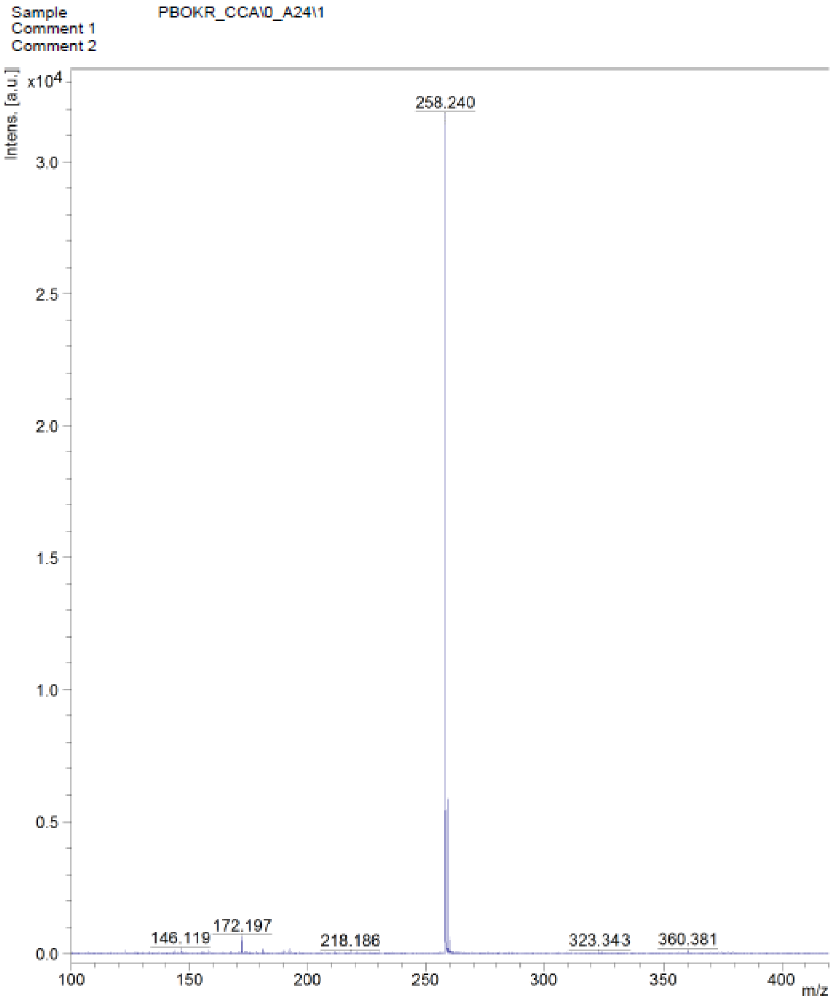

Figure S44. MALDITOF MS spectrum of *N*-[(2*R*,3*S*)-(3-hydroxyoxolan-2-yl)methyl]-*N*,*N*-dimethyl-*N*-octylammonium tosylate (**5c**), m/z 258,240 ([M-OTs]$^+$).

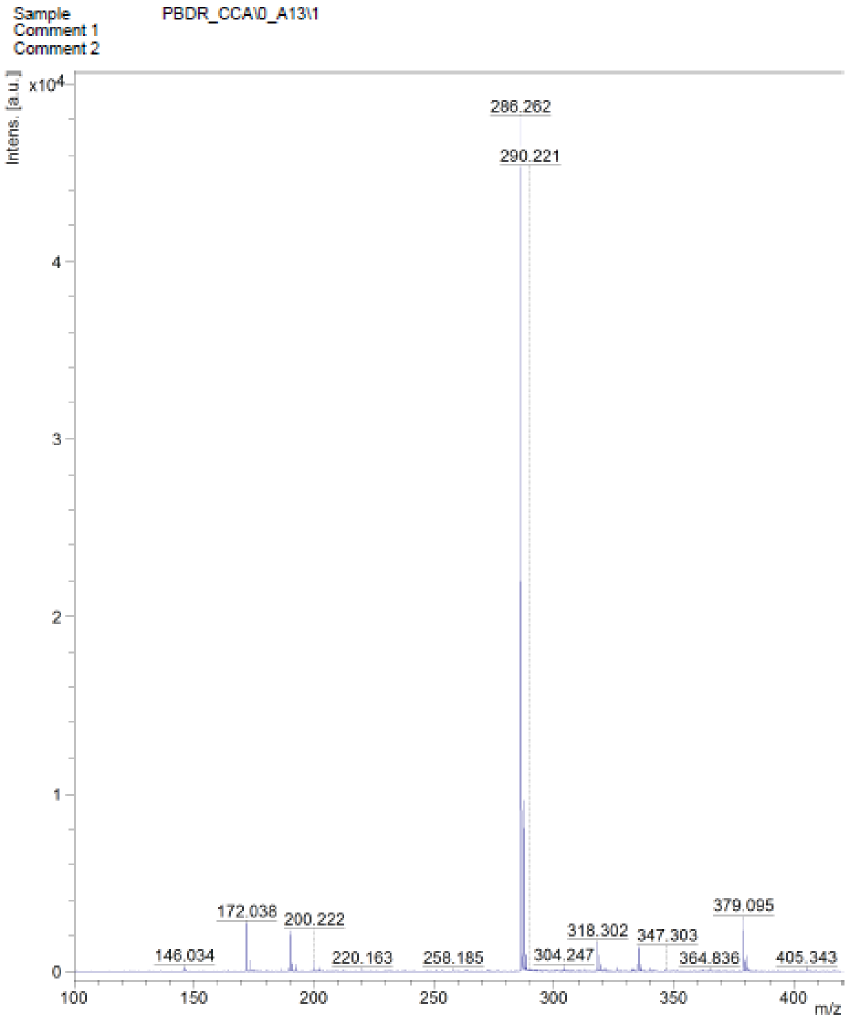

Figure S45. MALDITOF MS spectrum of *N*-[(2*R*,3*S*)-(3-hydroxyoxolan-2-yl)methyl]-*N*-decyl-*N*,*N*-dimethylammonium tosylate (**5d**) m/z 286,262 ([M-OTs]$^+$).

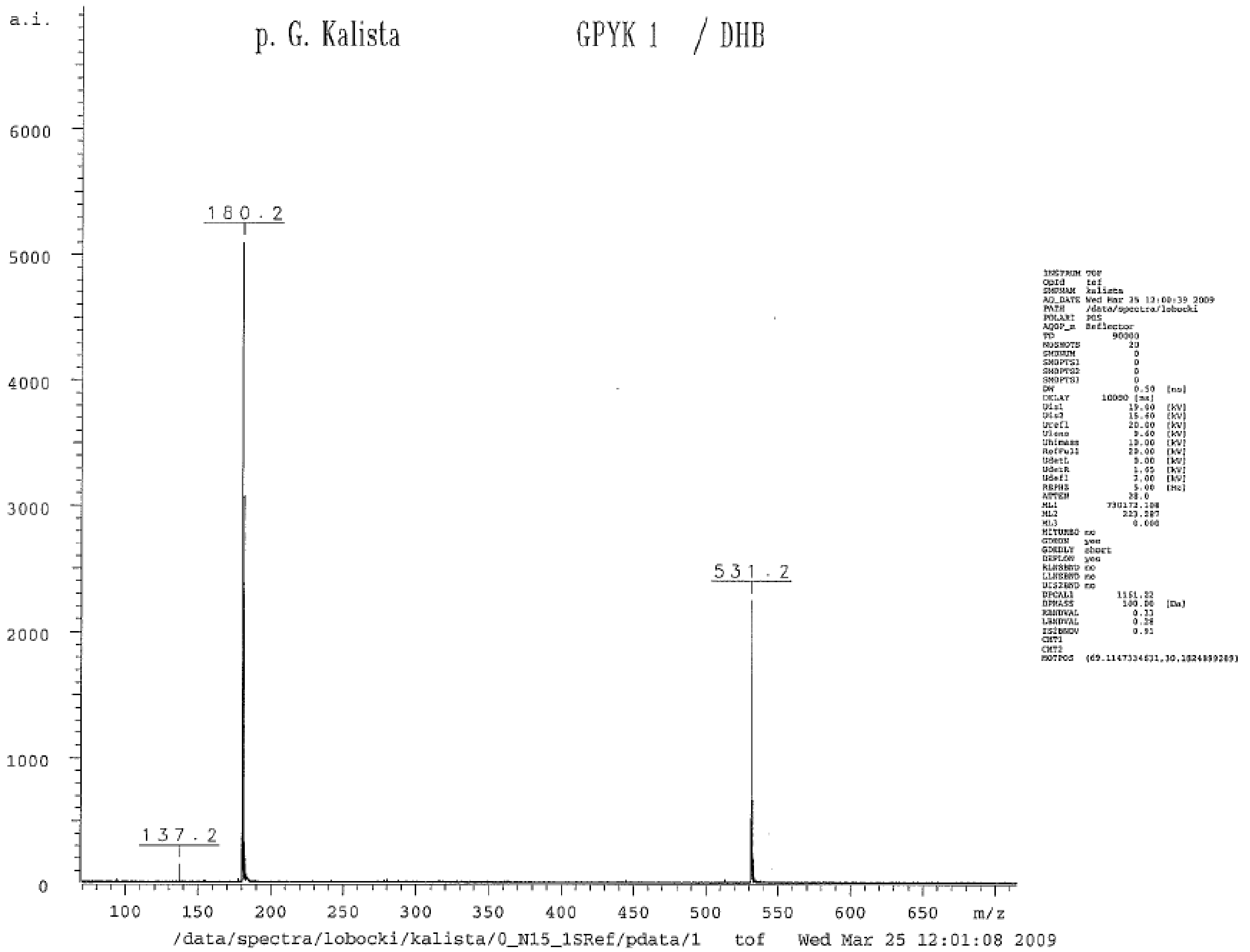

Figure S46. MALDITOF MS spectrum of *N*-[(2*R*,3*S*)-(3-hydroxyoxolan-2-yl)methyl]pyridinium tosylate (**5e**), m/z 180,20 ([M-OTs]$^+$).

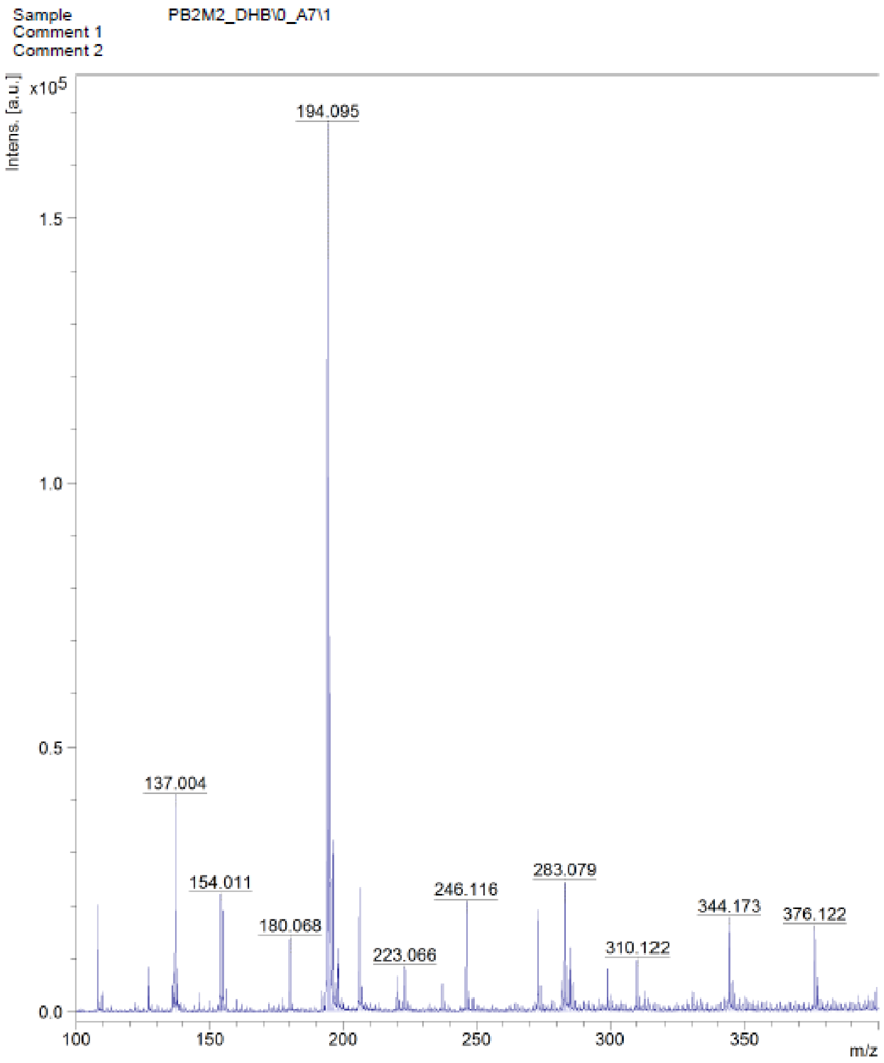

Figure S47. MALDITOF MS spectrum of *N*-[(2*R*,3*S*)-(3-hydroxyoxolan-2-yl)methyl]-2-methylpyridinium tosylate (**5f**) m/z 194,095 ([M-OTs]$^{+}$).

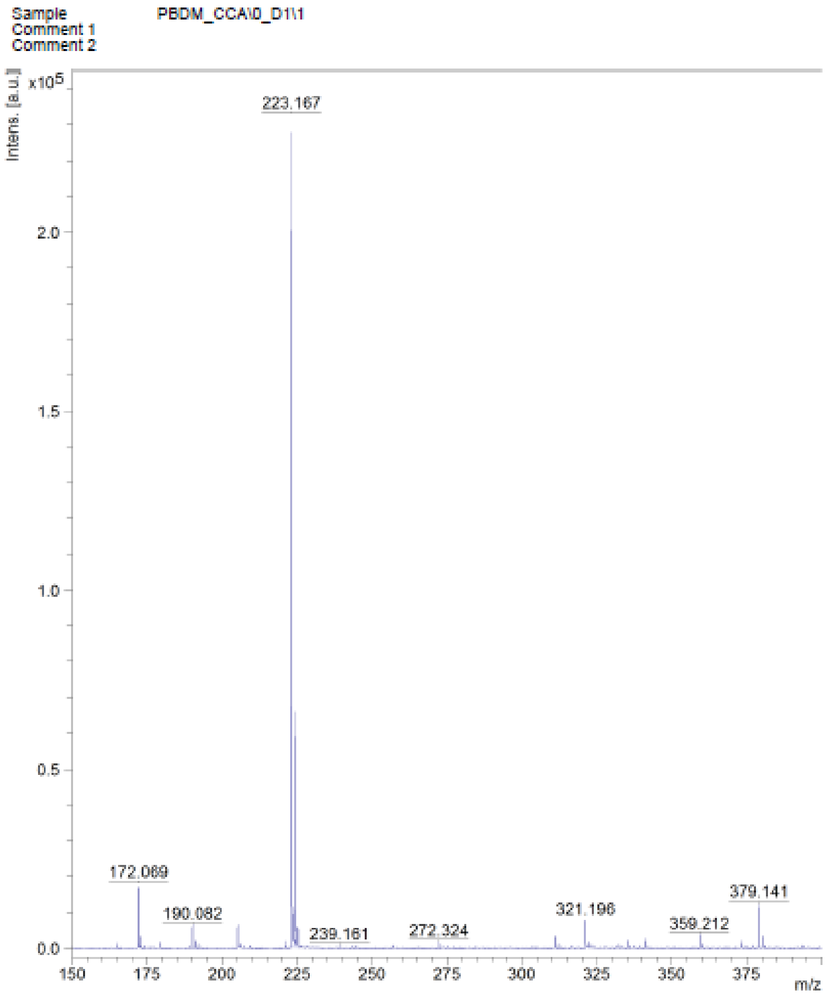

Figure S48. MALDITOF MS spectrum of *N*-[(2*R*,3*S*)-(3-hydroxyoxolan-2-yl)methyl]-4-(*N*,*N*-dimethylamino)pyridinium tosylate (**5g**), m/z 223,167 ($[M\text{-}OTs]^+$).

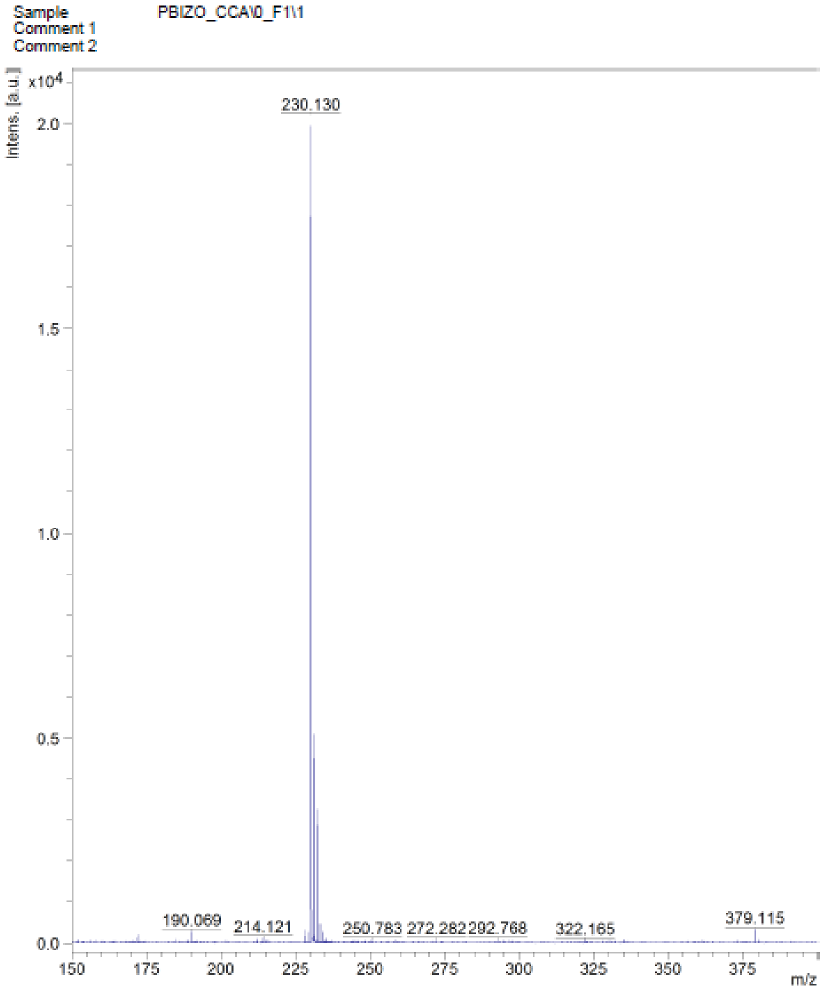

Figure S49. MALDITOF MS spectrum of *N*-[(2*R*,3*S*)-(3-hydroxyoxolan-2-yl)methyl]isoquinolinium tosylate (**5h**) m/z 230,130 ([M-OTs]$^+$).

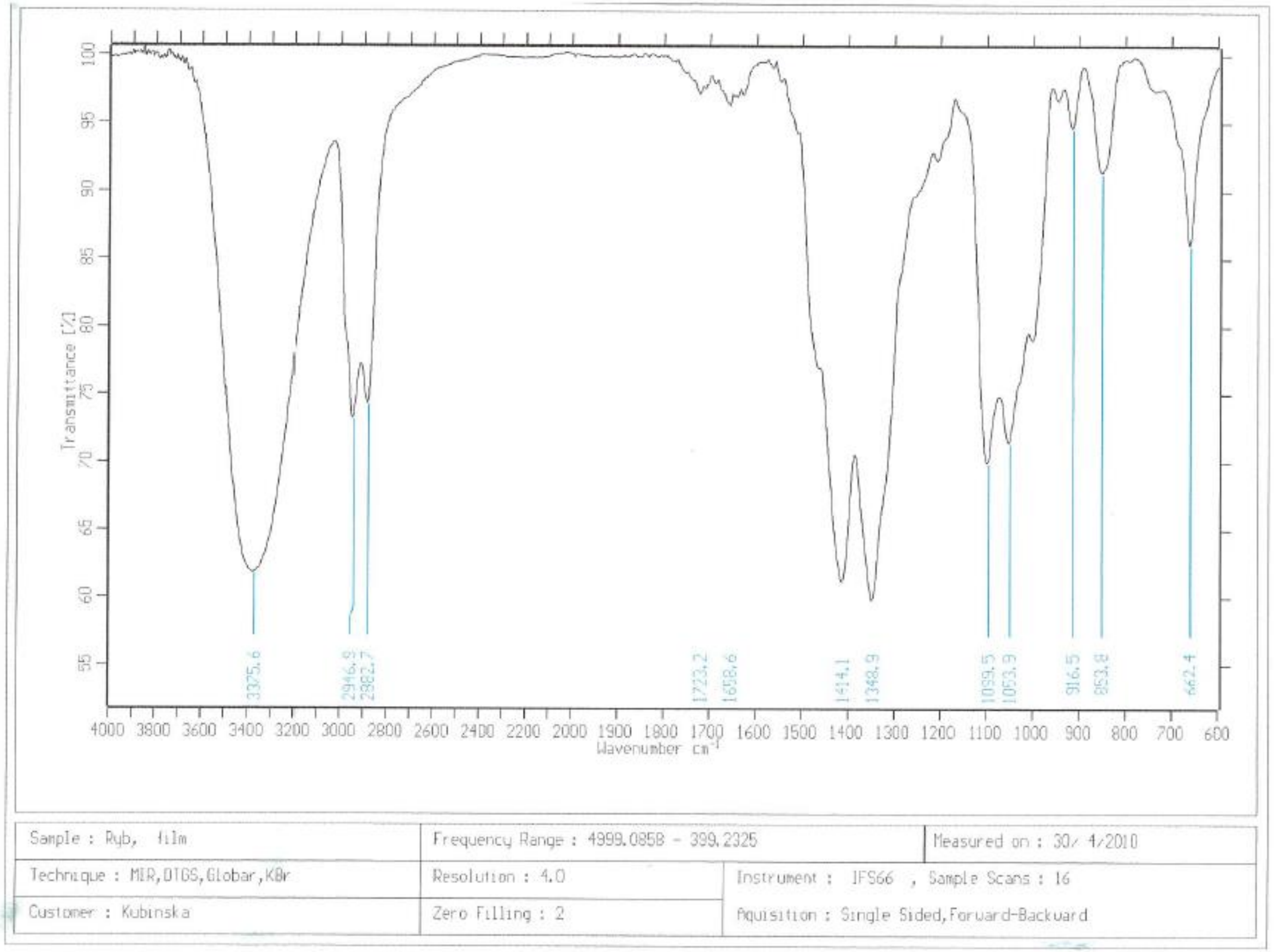

Figure S50. IR spectrum of (2*R*,3*S*)-2-(hydroxymethyl)oxolan-3-ol (**3**).

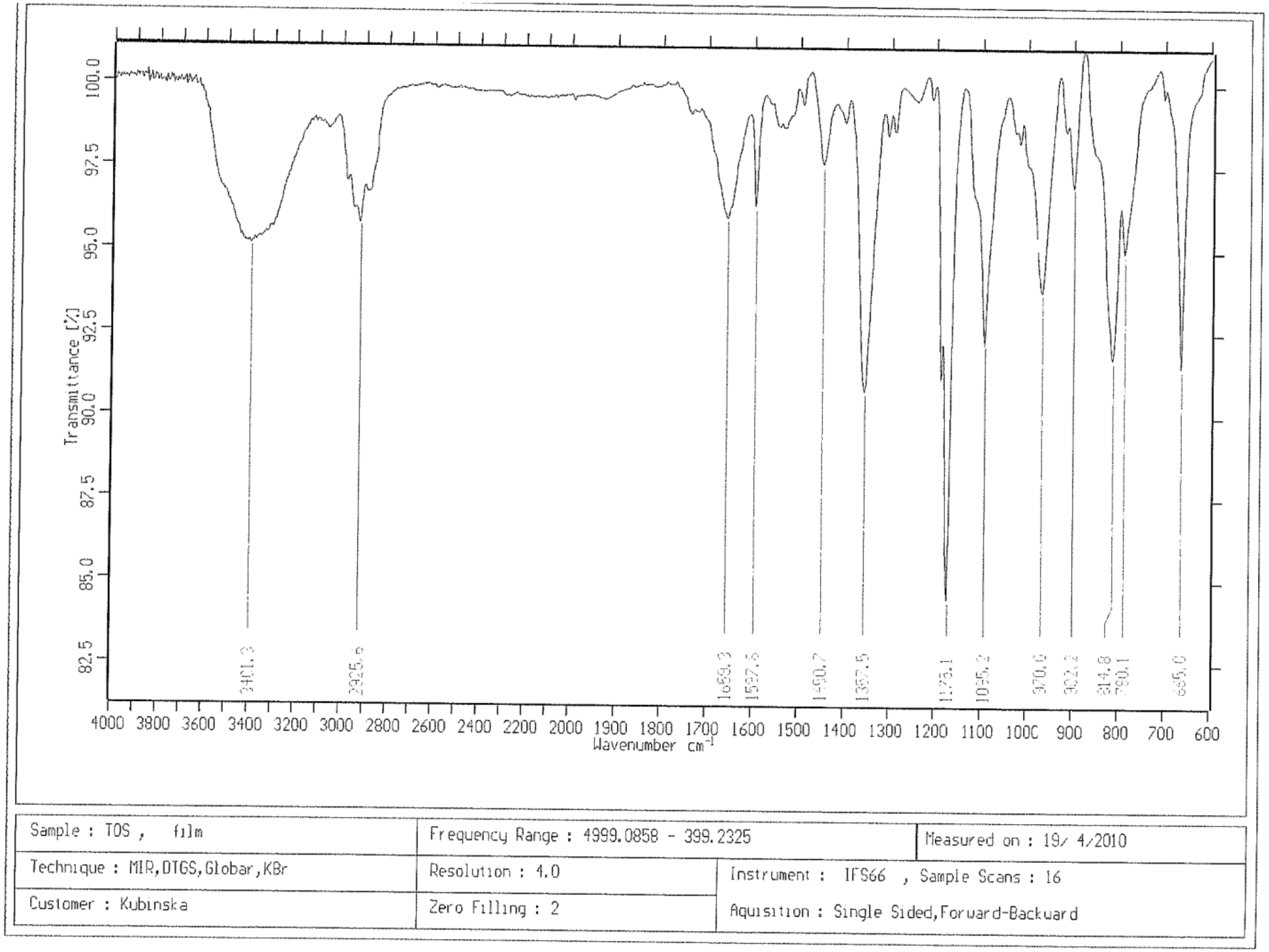

Figure S51. IR spectrum of (2*R*,3*S*)-2-(*O*-tosylmethoxyl)oxolan-3-ol (**4**).

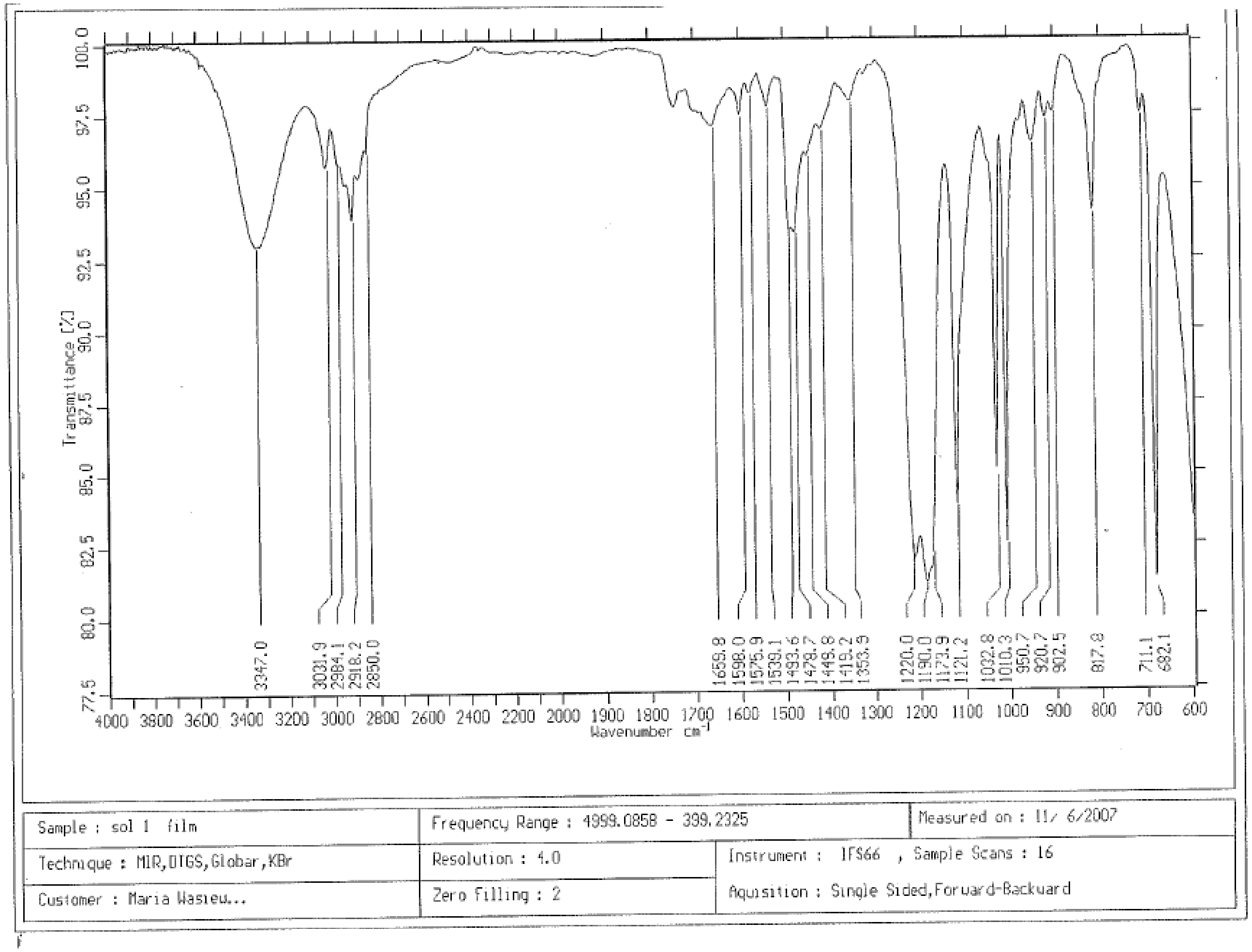

Figure S52. IR spectrum of *N*-[(2*R*,3*S*)-(3-hydroxyoxolan-2-yl)methyl]-*N*,*N*,*N*-trimethylamonium tosylate (**5a**).

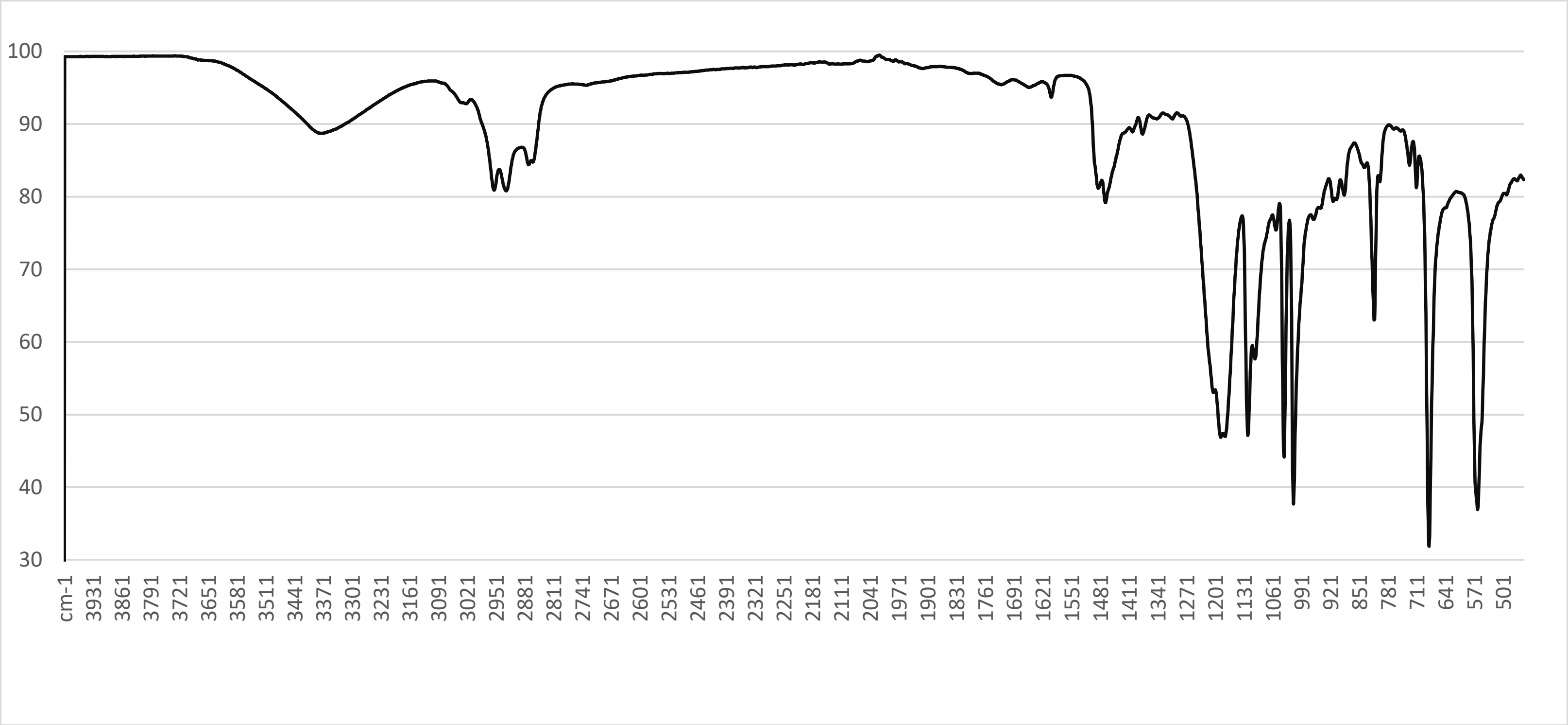

Figure S53. IR spectrum of *N*-[(2*R*,3*S*)-(3-hydroxyoxolan-2-yl)methyl]-*N*-hexyl-*N*,*N*-dimethylammonium tosylate (**5b**).

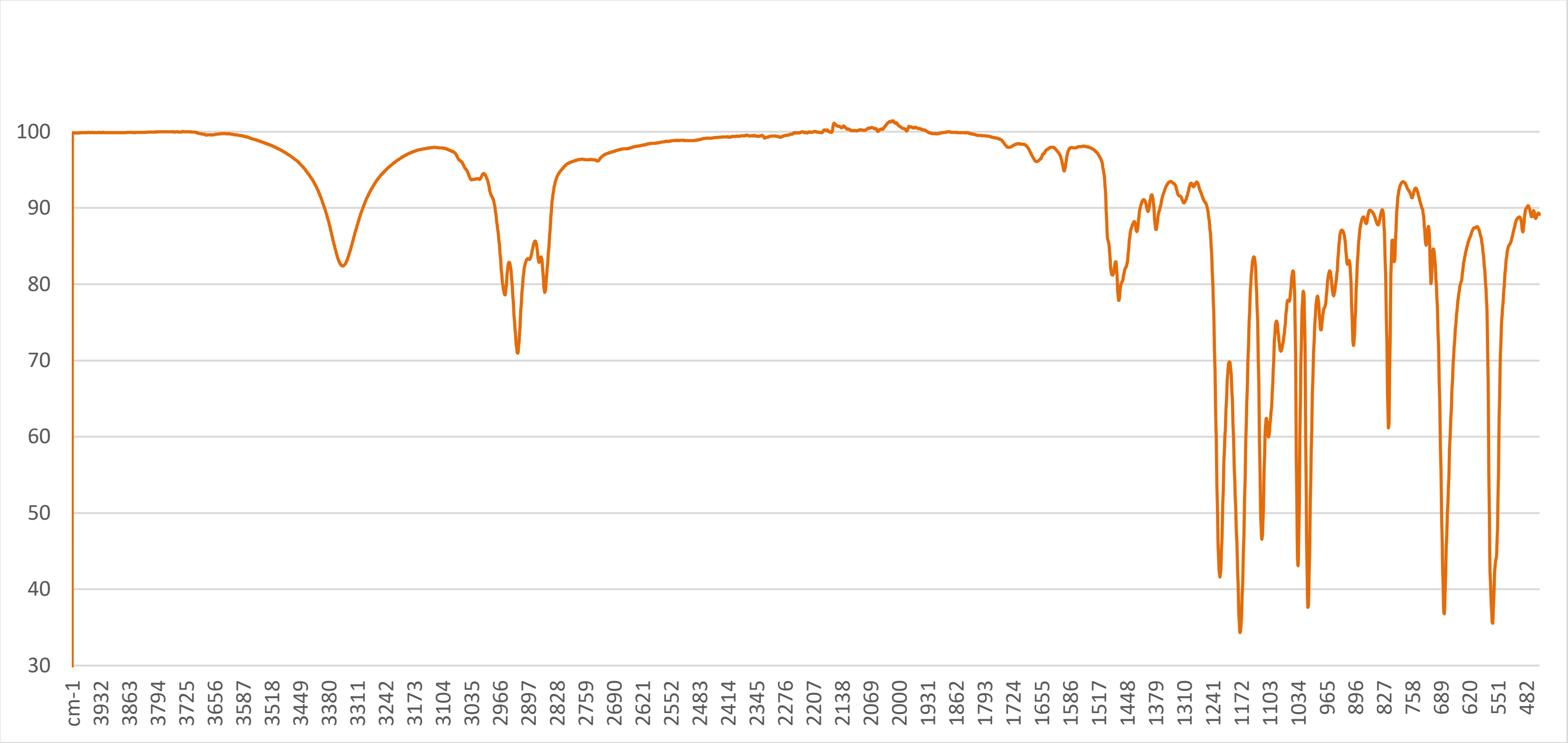

Figure S54. IR spectrum of *N*-[(2*R*,3*S*)-(3-hydroxyoxolan-2-yl)methyl]-*N*,*N*-dimethyl-*N*-octylammonium tosylate (**5c**).

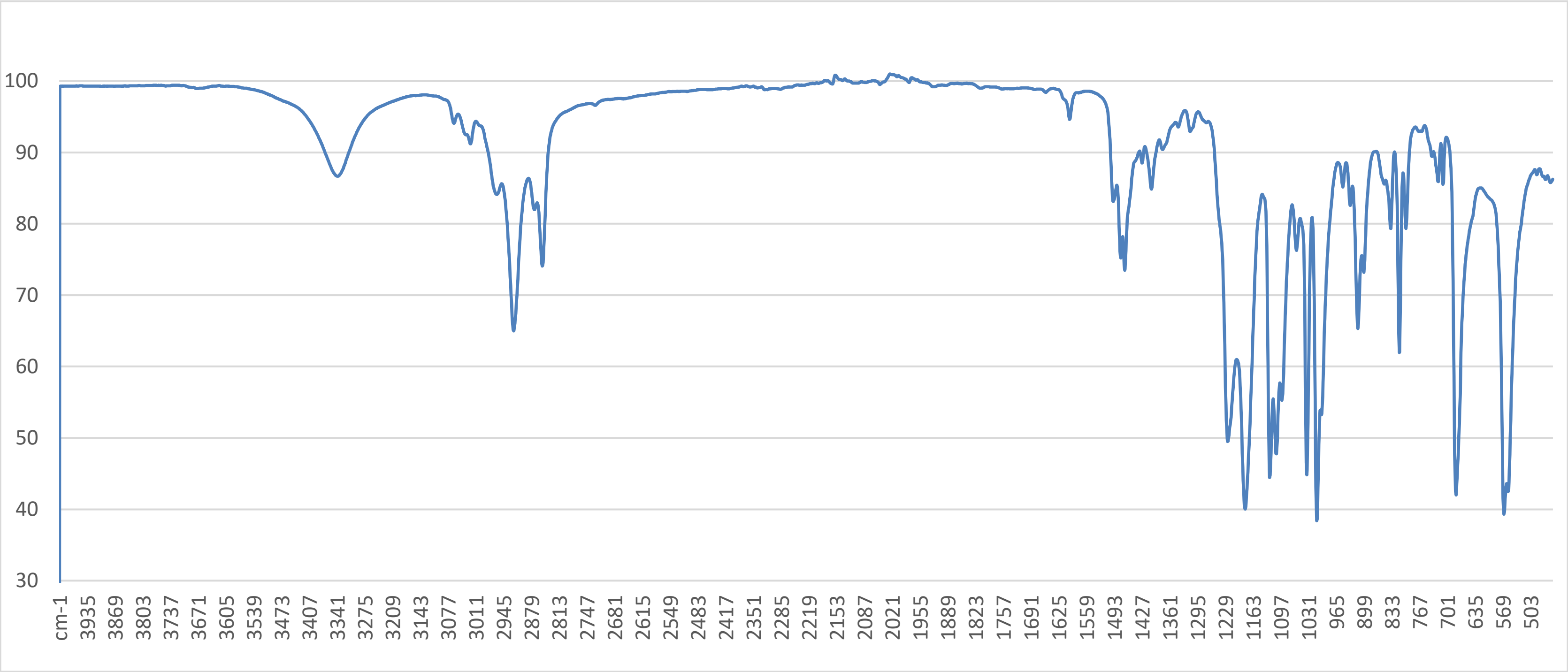

Figure S55. IR spectrum of *N*-[(2*R*,3*S*)-(3-hydroxyoxolan-2-yl)methyl]-*N*-decyl-*N*,*N*-dimethylammonium tosylate (**5d**).

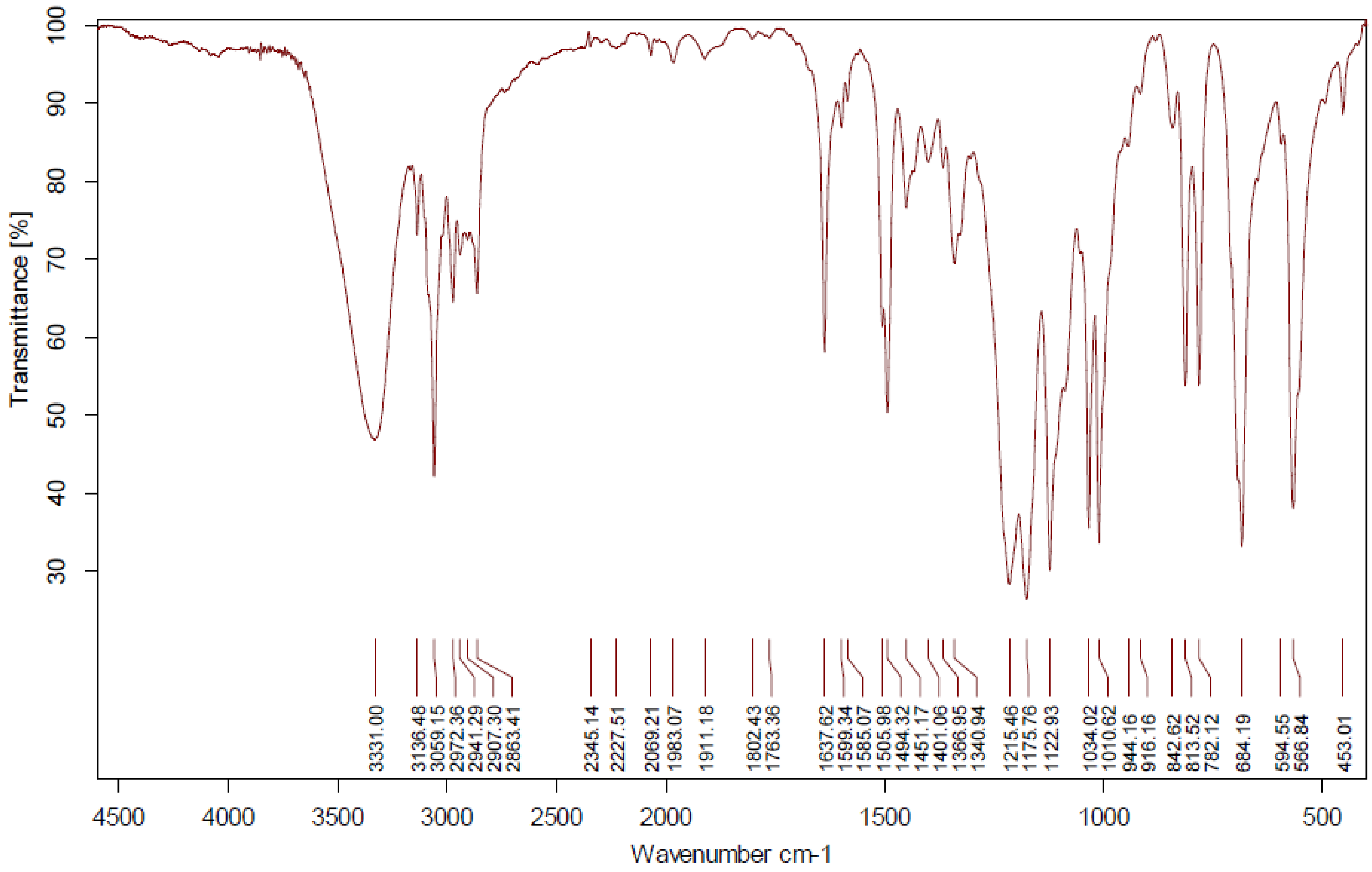

Figure S56. IR spectrum of *N*-[(2*R*,3*S*)-(3-hydroxyoxolan-2-yl)methyl]pyridinium tosylate (**5e**).

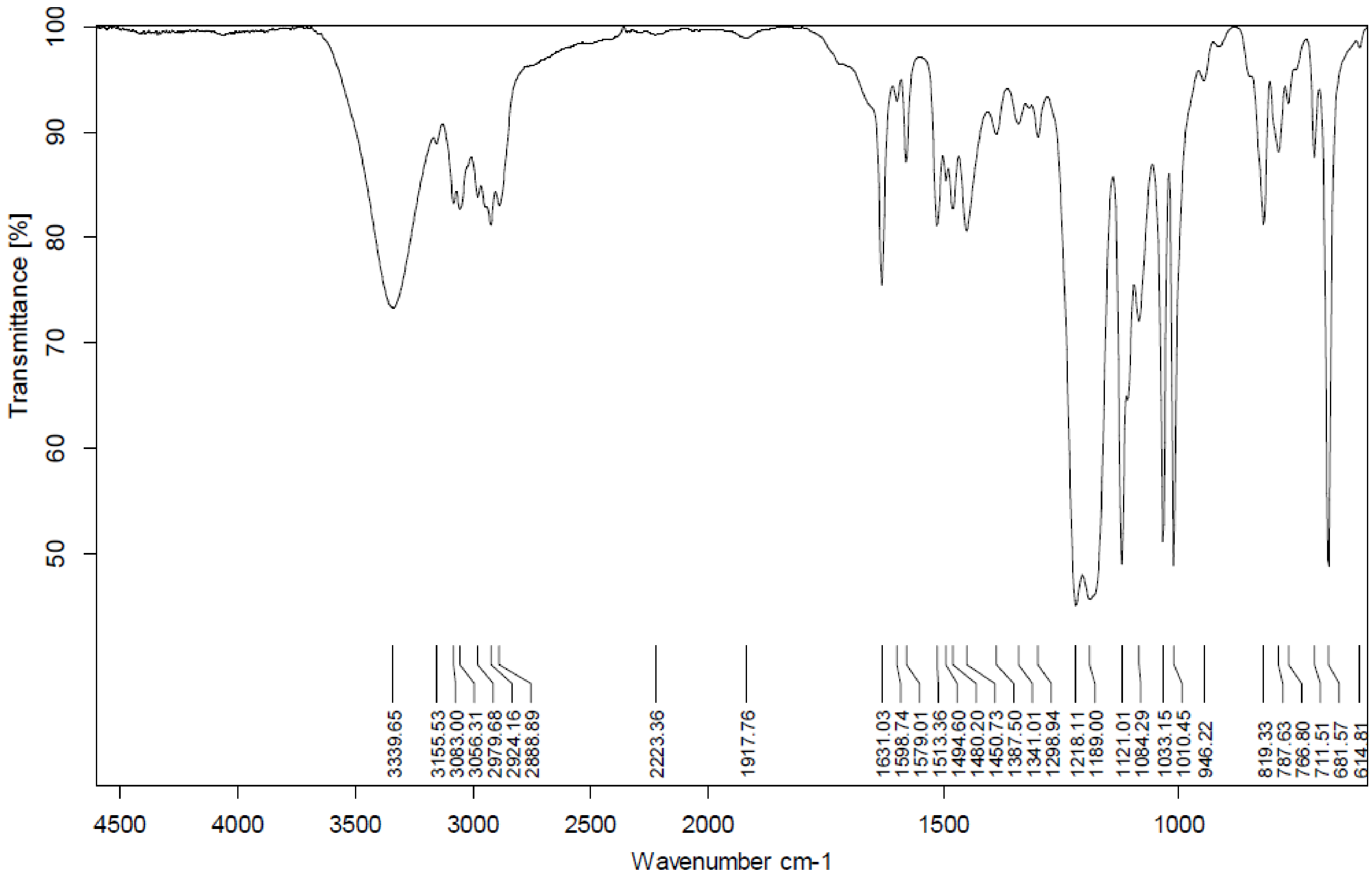

Figure S57. IR spectrum of *N*-[(2*R*,3*S*)-(3-hydroxyoxolan-2-yl)methyl]-2-methylpyridinium tosylate (**5f**).

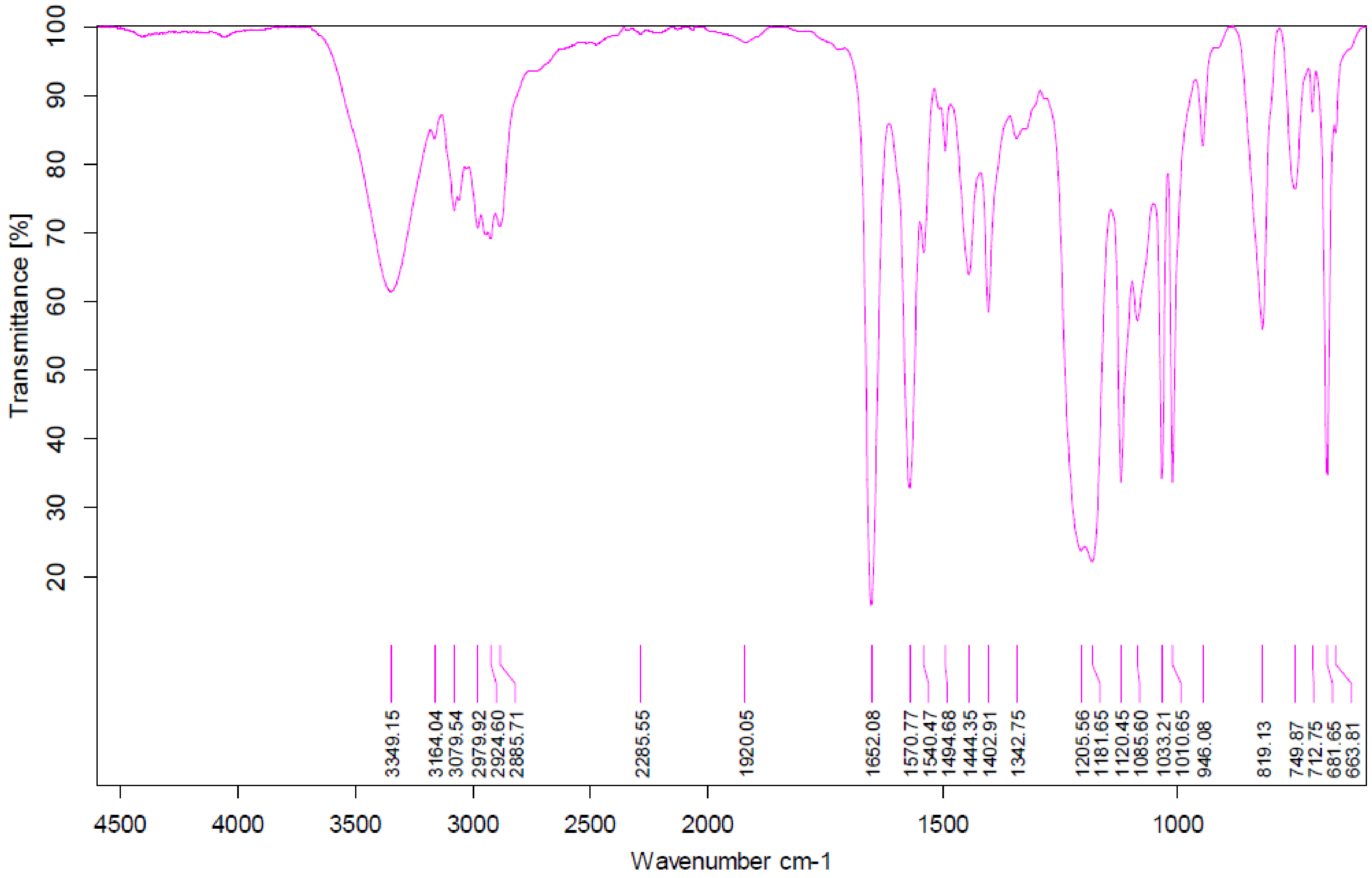

Figure S58. IR spectrum of *N*-[(2*R*,3*S*)-(3-hydroxyoxolan-2-yl)methyl]-4-(*N*,*N*-dimethylamino)pyridinium tosylate (**5g**).

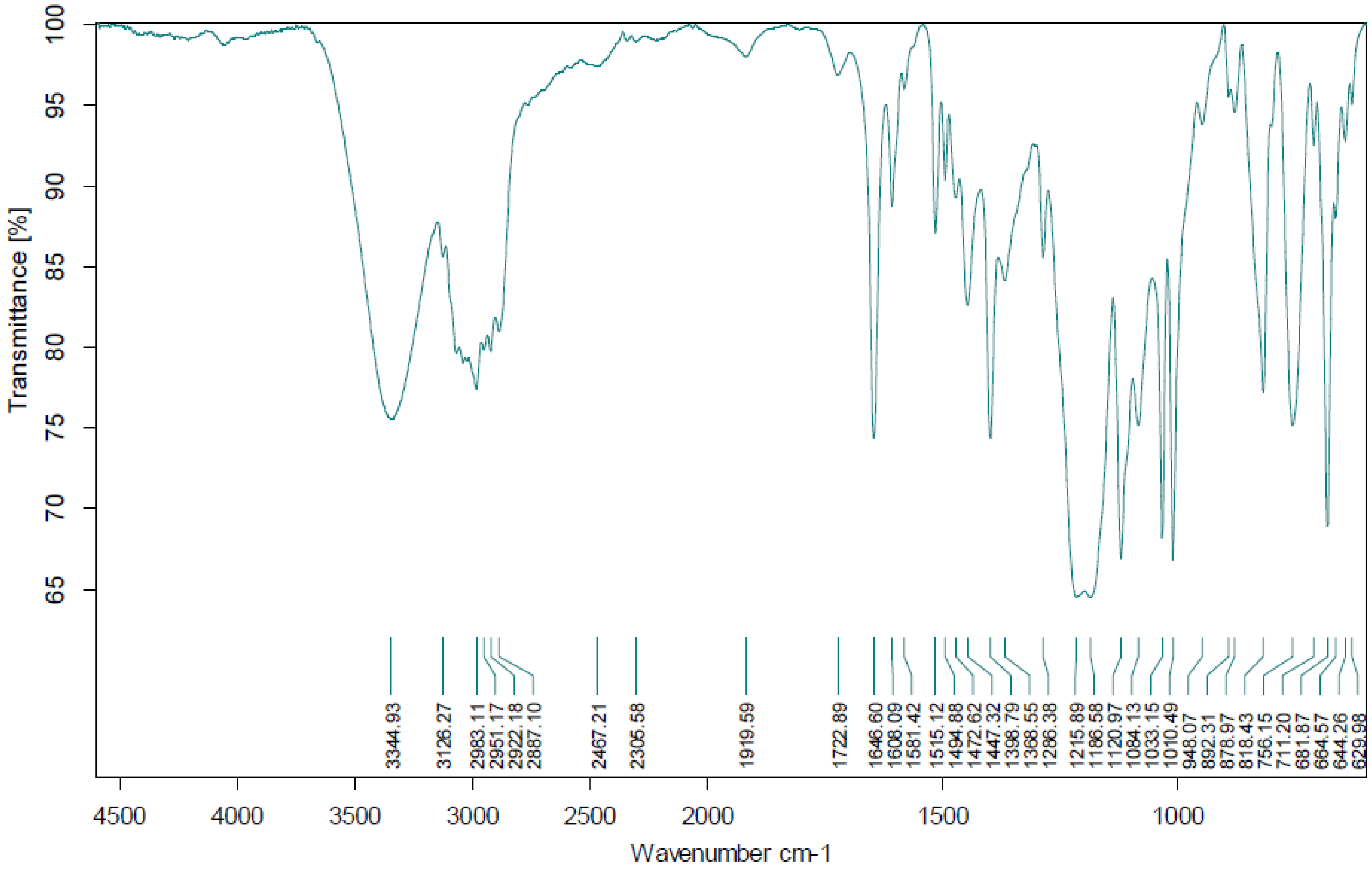

Figure S59. IR spectrum of *N*-[(2*R*,3*S*)-(3-hydroxyoxolan-2-yl)methyl]isoquinolinium tosylate (**5h**).
